# Evaluation of *Lactiplantibacillus plantarum* CRS 33 to therapeutic effects on a murine model of *Escherichia coli*-induced endometritis

**DOI:** 10.3389/fvets.2025.1608791

**Published:** 2025-10-31

**Authors:** Mingchao Liu, Xiangfu Wen, Mingque Feng, Yan Sun, Xiaowei Feng, Tianxiong Jin, Bei Liu, Shahid Muhammad, Kangping Liu, Jia Cheng, Jianguo Li

**Affiliations:** ^1^College of Animal Science and Technology, Hebei Agricultural University, Baoding, China; ^2^Key Laboratory of Healthy Breeding in Dairy Cattle (Co-Construction by Ministry and Province), Ministry of Agriculture and Rural Affairs, Hebei Agricultural University, Baoding, China; ^3^College of Veterinary Medicine, Hebei Agricultural University, Baoding, China; ^4^Center of Microbiology and Biotechnology, Veterinary Research Institute, Peshawar, Pakistan

**Keywords:** *Lactiplantibacillus plantarum* CRS33, *Escherichia coli*, whole-genome, inflammatory factors, bovine endometritis, probiotic therapy, microbiome, murine model

## Abstract

**Introduction:**

Bovine endometritis is a common postpartum uterine disease in dairy cows that is traditionally treated with antibiotics. However, excessive antibiotic use can lead to antimicrobial resistance and treatment failure. *Lactiplantibacillus plantarum* CRS33, a novel probiotic strain isolated from the uterus of a healthy cow, exhibits strong antibacterial potential. This study aimed to investigate the probiotic characteristics of *Lactiplantibacillus plantarum* CRS33 through whole-genome sequencing and to evaluate its anti-inflammatory effects in a mouse model of *Escherichia coli*–induced endometritis.

**Methods:**

Whole-genome sequencing was performed to identify genes related to antibacterial, anti-inflammatory, and immune-regulatory activities, and to confirm the absence of antibiotic resistance and virulence genes. Female mice were induced with *Escherichia coli* endometritis and treated with *Lactiplantibacillus plantarum* CRS33 at a dose of 1 × 10^⁹^ CFU/mL. Uterine morphology, wet weight index, inflammatory cell infiltration, cytokine levels (IL-6, IL-1β, IL-8, TNF-α), and uterine microbiota composition were analyzed.

**Results:**

Genomic analysis revealed that *Lactiplantibacillus plantarum* CRS33 contains multiple functional genes related to antimicrobial, anti-inflammatory, and immune-modulatory pathways and lacks antibiotic resistance or pathogenic determinants. Treatment with *Lactiplantibacillus plantarum* CRS33 significantly alleviated uterine inflammation, reduced the wet weight index (*p* < 0.05), and improved histopathological lesions. It also decreased pro-inflammatory cytokine levels and inflammatory cell infiltration, while enhancing microbial diversity and increasing the abundance of beneficial bacterial taxa.

**Discussion:**

*Lactiplantibacillus plantarum* CRS33 demonstrates strong anti-inflammatory and microbiota-regulating properties in *Escherichia coli*–induced endometritis, highlighting its potential as a safe and effective probiotic alternative to antibiotics. Further validation in dairy cows is warranted to confirm its therapeutic potential under practical conditions.

## 1 Introduction

Bovine endometritis, a prevalent postpartum condition in dairy cows, adversely affects the receptivity and structural integrity of the endometrium (1). This condition potentially impairs embryo implantation, increases vulnerability to recurrent miscarriages (2, 3), and elevates the risks of infertility and pregnancy-related complications (4, 5). The incidence of bovine clinical endometritis has increased significantly as dairy farming has been scaled up worldwide, resulting the enormous economic losses of dairy farming (6). In China, the occurrence rate of endometritis can reach 35–45% (7), while the positive rate of bovine endometritis was found in Canada (13–64%) (8), New Zealand (27%) (9), and America (4.8–52.6%) (10).

*Escherichia coli* is the primary culprit responsible for causing clinical bovine endometritis (11). The odds of isolating *E. coli* from the uterus of cows with endometritis were as high as 67%, significantly higher than in healthy cows (12). The management of bovine endometritis primarily focuses on antibiotic treatment. However, the extensive and improper use of antibiotics in treating bovine endometritis has resulted in serious issues of antibiotic resistance, which has raised concerns about the food safety (13–15). Therefore, finding a safe and effective alternative to antibiotics for treating and preventing bovine endometritis, while reducing residual contamination, has become a crucial objective in dairy cow breeding programs.

Probiotics can exert protective effects through self-adherence, production of biosurfactants and hydrogen peroxide, and modulation of the host innate immune system (16). Numerous studies have demonstrated that lactic acid bacteria (LAB) possess therapeutic potential in combating uterine inflammation (17, 18). For example, lactobacilli supplementation has been associated with maintaining vaginal microbial balance (15) and attenuating *E. coli*-induced inflammatory responses in bovine endometrium (19–21). More recent investigations (2022–2024) further confirm that specific strains such as *Lactobacillus rhamnosus* GR-1 and engineered *Lactobacillus johnsonii* can significantly downregulate pro-inflammatory cytokines and improve uterine health in both *in vitro* and *in vivo* models (18, 22, 23). Probiotic consortia isolated from the uterine tract of buffaloes have also shown promising antimicrobial and immunomodulatory effects (24). However, these benefits appear to be strain-dependent, and their efficacy has not been consistently validated in bovine clinical settings, leaving uncertainty regarding their practical application.

*Lactiplantibacillus plantarum* CRS33, a probiotic isolated from the intravaginal tract of fit cows, demonstrated significant efficacy in suppressing the growth of *E. coli.* In addition, *Lactiplantibacillus plantarum* CRS33 was sensitive to a wide range of antibiotics (Ampicillin, Tetracycline, Erythromycin, Trimethoprim, Chloramphenicol, Penicillin) and did not negatively affect the physiological activity of mice. Given the diverse range of biological activities exhibited by this bacterium *in vitro*, it is crucial to gain a deeper understanding of *Lactiplantibacillus plantarum* CRS33, as well as its role in treating endometritis. To further clarify its therapeutic potential, we conducted whole-genome sequencing to characterize the genetic basis of its antibacterial and immunomodulatory properties, and we evaluated its efficacy in a murine model of *E. coli*-induced endometritis.

The mouse model offers a practical and economical system for assessing probiotic therapies in bovine endometritis, given its feasibility and immune similarities to cattle (24, 25). Recent studies further support its use in evaluating probiotic interventions against uterine tract inflammation (26, 27), forming the basis for our investigation of *Lactiplantibacillus plantarum* CRS33 in *E. coli*-induced endometritis.

## 2 Materials and methods

### 2.1 Bacterial strains

Uterine tract secretions were collected from the cervical region of 10 healthy dairy cows (25–50 days postpartum) at a dairy farm located in Mancheng District, Baoding City, Hebei Province, China. The samples were fully enriched, and 100 μL of the enriched sample was spread on MRS medium (AOBOX, Beijing, China). The plates were sealed and incubated anaerobically at 37 °C for 24 h. White, pinpointed single colonies were selected for purification, and the purification process was carried out according to Berger’s manual for bacterial identification (28). After the purification process was completed, *Lactiplantibacillus plantarum* CRS33 was successfully isolated and obtained. The culture concentration of 1 × 10^9^ CFU/mL was determined using a stepwise dilution method.

The *E. coli* strain (O111: K58, B4; CVCC1450) was provided by the Laboratory of Clinical Nutrition and Immunology, affiliated with the College of Veterinary Medicine, China Agricultural University, Beijing. This strain was incubated in LB broth (AOBOX, Beijing, China) at 37 °C under 180 rpm orbital shaking for a duration of twelve hours, and its bacterial density was calibrated to 1 × 10^10^ CFU/mL.

### 2.2 Whole-genome sequencing of *Lactiplantibacillus plantarum* CRS33

*Lactiplantibacillus plantarum* CRS33 was cultured in MRS broth at 37 °C for 24 h. The cells were then collected through centrifugation at 12,000 × *g* for two minutes. Genomic DNA was isolated from the harvested cell pellets using the EasyPure^®^ Bacteria Genomic DNA Kit (TransGen, Beijing, China). The purity and concentration of the DNA were measured at a wavelength of 260 nm with the NanoDrop^®^ One Spectrophotometer (NanoDrop Technologies, Wilmington, DE, United States). To minimize contamination, blank extraction controls (without bacterial cells) were included during DNA extraction, and negative controls were also processed during amplification and library preparation.

In this study, long-read sequencing was conducted with the Nanopore sequencer (Oxford Nanopore Technologies, United Kingdom), and short-read sequencing was performed on the Illumina platform using the NEBNext^®^ Ultra™ DNA Library Prep Kit (Ipswich, MA, United States) with a DNA input amount of 1 μg and insert sizes ranging from 10 to 20 kb. The sequencing data were integrated using a hybrid assembly approach, with support from Biomarker Technologies Co., Ltd. (Beijing, China). The bacterial genome assembly and polishing were carried out using Canu (v1.5) (29), followed by error correction via Pilon (v1.22) (30) and Racon (v3.4.3) (31). Finally, the contigs were merged to produce a complete genomic sequence. Quality control of sequencing included removal of adapter sequences, filtering of low-quality reads, and verification of assembly accuracy by comparing read mapping rates and genome coverage.

The annotation of bacterial genomic data was performed using several comprehensive databases, including the Non-Redundant Protein (NR) Sequence Database (32), Swiss-Prot (33), Pfam Protein Families Database (34), the evolutionary genealogy of genes framework: Non-supervised Orthologous Groups (eggNOG) (35), Gene Ontology (GO) (36), and the Kyoto Encyclopedia of Genes and Genomes (KEGG) (37). Through this annotation process, candidate genes associated with the probiotic functionality of *Lactiplantibacillus plantarum* CRS33 were identified using a suite of bioinformatics tools. A circular genomic map was generated with CGView software, and the complete genome sequence was submitted to the NCBI database with the accession identifier CP125654.1.

### 2.3 Animals and experimental design

SPF-grade female KM mice, aged between 6 and 8 weeks, were sourced from Specific Pathogen-Free (SPF) Beijing Biotechnology Co. and housed in groups within the animal care facility of Hebei Agricultural University. The animals were maintained on a standardized diet with unrestricted access to food and water. Bedding materials and hygienic conditions were routinely monitored and upheld. The mice were acclimatized to the experimental environment for a period of 7 days prior to the commencement of the study. we monitored the estrous cycle of all mice during the study. All mice were sacrificed and sampled during the non-estrous phase of the cycle to minimize any potential hormonal fluctuations that could influence uterine physiology and microbiota. The handling of animals and experimental protocols adhered to the ethical guidelines approved by the Institutional Animal Care and Use Committee (IACUC) of Hebei Agricultural University (Approval Number: 2021041).

**Figure 1:**
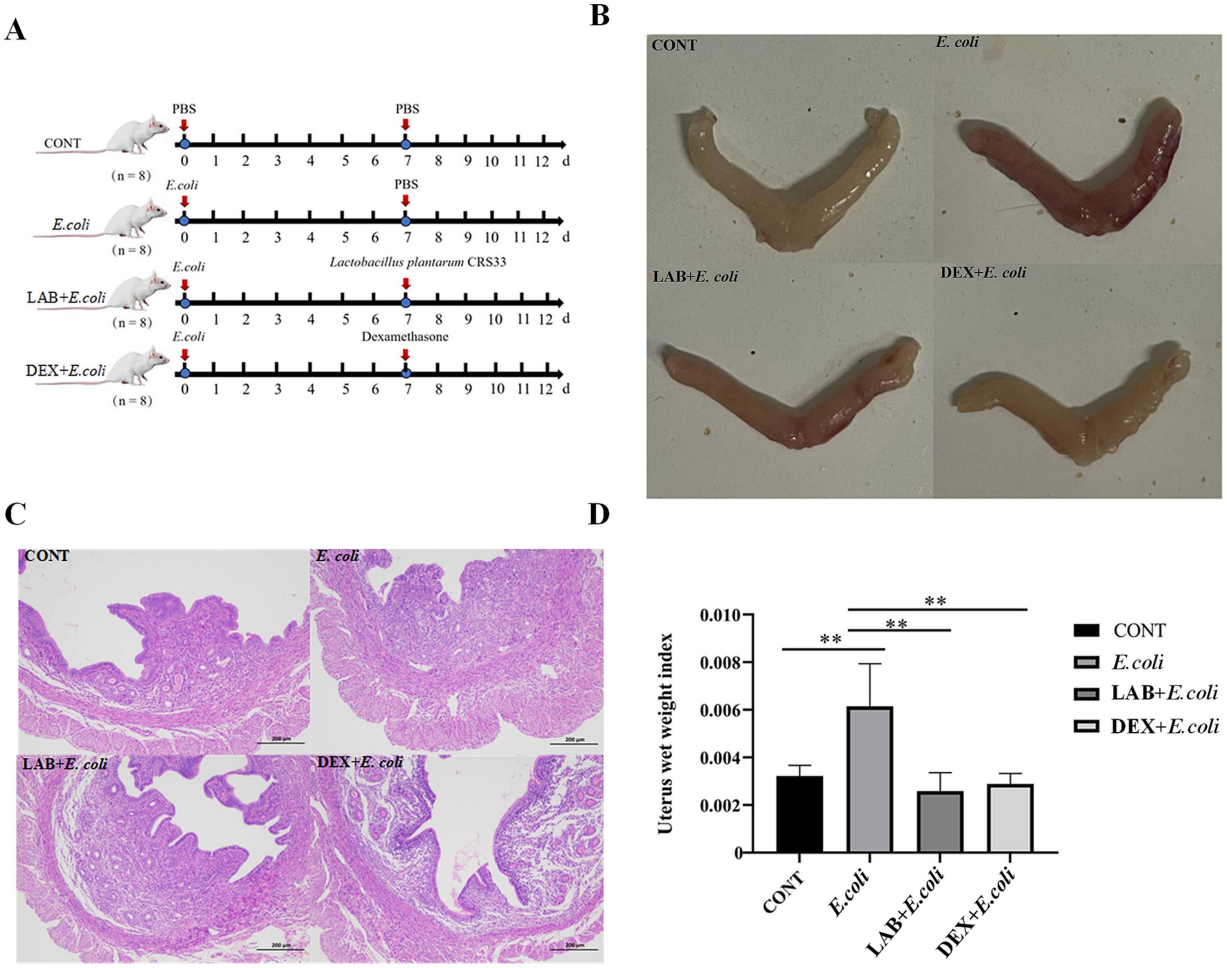
Effect of *Lactiplantibacillus plantarum* CRS33 on uterine lesions caused by *E. coli*. **(A)** Experimental scheme **(B)** mouse uterus morphology; **(C)** Mice uterine tissue sections (100×); **(D)** The uterine index was calculated for each group based on uterine weight and mouse weight. *p < 0.05, **p < 0.01.

Thirty-two mice were randomly assigned to four experimental groups: the control group (CONT), the *E. coli* group (*E. coli*), the *Lactiplantibacillus plantarum* CRS33 treatment group (LAB+*E. coli*), and the dexamethasone treatment group (DEX + *E. coli*). Each group consisted of 8 mice, and group assignments were conducted to ensure balanced experimental conditions (Figure 1A). Experimental groups (*E. coli*, LAB+*E. coli*, and DEX + *E. coli*) received a daily uterine infusion of 0.2 mL of 1 × 10^10^ CFU/mL *E. coli* solution from days 1 to 6. The LAB+*E. coli* group was subjected to uterine infusion 0.2 mL 1 × 10^9^ CFU/mL *Lactiplantibacillus plantarum* CRS33 and DEX + *E. coli* group was intraperitoneal injection 0.2 mL 5 mg/kg dexamethasone daily from days 7 to 12, respectively. CONT group was administered 0.2 mL 0.9% saline solution, serving as negative control. All mice were euthanized on day 13 after completion of treatments. Blood samples were first collected via orbital (eye) bleeding for serum cytokine analysis, followed by immediate removal of uterine tissues. The tissues were dissected in a standardized order (left horn to right horn) and either fixed in 4% paraformaldehyde for histological analysis or snap-frozen in liquid nitrogen for molecular assays, ensuring consistency and methodological rigor.

### 2.4 Histopathological examination

Uterine tissues from all experimental groups were harvested and preserved in 4% paraformaldehyde for 24 h. The samples underwent thorough washing and dehydration through a standardized protocol utilizing sterile water and graded ethanol solutions. Subsequently, tissue sections with a thickness of 4 μm were prepared and subjected to hematoxylin and eosin staining. The stained sections were meticulously examined under a light microscope (NIKON, Tokyo, Japan) at a magnification of ×100 to evaluate the uterine tissue morphology. All histopathological evaluations were conducted in a blinded manner by investigators who were unaware of the group assignments.

### 2.5 Immunohistochemical analysis

For immunohistochemical analysis, paraffin-embedded slides were deparaffinized using xylene and a series of graded alcohols (Shanghai Leica Instrument Co., Ltd.). Antigen retrieval was performed by immersing the sections in a sodium citrate buffer solution (0.01 M, pH 6.0) at 95 °C for 5 min. Endogenous peroxidase activity was quenched by incubating the slides in 3% hydrogen peroxide (H₂O₂) for 10 min at room temperature. Following this, the sections were incubated overnight at 4 °C with primary antibodies, including CD45 (1:500, Servicebio, Wuhan, China), CD68 (1:500, Servicebio, Wuhan, China), and MPO (1:1000, Servicebio, Wuhan, China). After three washes, the sections were treated with a secondary antibody (HRP-labeled goat anti-rabbit, 1:1000, ZS-Bio, Beijing, China) at 37 °C for 20 min. Finally, the slides were subjected to diaminobenzidine (DAB) staining and observed under a microscope (NIKON, Tokyo, Japan). Immunohistochemical analyses were also evaluated independently by blinded observers, with group allocation concealed throughout the assessment process to minimize bias.

### 2.6 ELISA for inflammatory cytokines in uterine tissues

The uterine tissues were homogenized in ice-cold phosphate-buffered saline (PBS) at a weight-to-volume ratio of 1:9. The homogenates were subsequently centrifuged at 12,000 rpm for 10 min at 4 °C, and the supernatants were carefully collected into sterile tubes. Inflammatory cytokines, including IL-1β, IL-6, IL-8, and TNF-*α*, were quantified using an ELISA kit (mlbio, S.h, China) following the manufacturer’s instructions.

### 2.7 Transcriptional gene expression of inflammation-related genes

Total RNA was isolated from uterine tissue and its concentration measured with a NanoDrop spectrophotometer (Thermo Fisher, United Kingdom). The cDNA synthesis process utilized the FastKing-RT SuperMix kit (Tiangen, Beijing). Quantitative real-time polymerase chain reaction (qRT-PCR) was conducted employing Tiangen’s qPCR reagents in conjunction with the LightCycler 480 system (Roche, Switzerland). The primers utilized for the experiments were designed with the aid of Primer-BLAST software, based on the coding sequence (CDS) regions of the target genes available within GenBank that are listed in Table 1.

**Table 1:** Primer sequences of target genes.

Target genes	Primers	
	Direction	Sequence (5′-3′)
IL-8	F	TGCATGGACAGTCATCCACC
	R	ATGACAGACCACAGAACGGC
IL-6	F	CTTCTTGGGACTGATGCTGGTGAC
	R	TCTGTTGGGAGTGGTATCCTCTGTG
IL-1β	F	CCTGGGCTGTCCTGATGAGAG
	R	TCCACGGGAAAGACACAGGTA
TNF-α	F	GGACTAGCCAGGAGGGAGAACAG
	R	GCCAGTGAGTGAAAGGGACAGAAC
GAPDH	F	CAATGTGTCCGTCGTGGATCT
	R	GTCCTCAGTGTAGCCCAAGATG

All treatments were analyzed in three independent experiments. Target mRNA values were normalized to GAPDH. The 2^−ΔΔCT^ method was applied for data analysis, and results were presented as the average fold change in mRNA expression levels in the experimental groups exposed to infection, compared to the non-infected control group (38).

### 2.8 16S rRNA

DNA was extracted from uterine samples obtained under sterile conditions using the TianGen DNA extraction kit (catalog number S30828-V2). The hypervariable V3–V4 regions of bacterial 16S rRNA genes were then amplified using the following primers: 338F: 5’-CCTACGGGAGGCAGCAG-3′ and 518R: 5’-ATTACCGCGGCTGCTGG-3′, and subjected to sequencing using the Illumina NovaSeq platform (Paisano Bio Ltd., S.h, China). High-quality sequencing data were generated, and analyses of alpha diversity (Chao1 and Shannon indices) and beta diversity were performed using the Personalbio Cloud Platform.^1^ For beta diversity analysis, the Bray-Curtis distance matrix was calculated (default setting), and PERMANOVA analysis was performed to assess between-group differences using the scikit-bio package in Python with 999 permutations. When the “adonis” test was applied, the vegan package in R was used to calculate the explained variance (R2) and significance (P) of the grouping scheme on the distance matrix, with 999 permutations.

### 2.9 Statistical analysis

Statistical analyses were performed using SPSS software version 21.0, employing one-way ANOVA. The results were expressed as the mean ± standard error of the mean (SEM) and further evaluated with GraphPad Prism version 7.0. Statistical significance was determined at a threshold of *p* < 0.05 for obvious differences and *p* < 0.01 for highly significant differences.

## 3 Results

### 3.1 Genomic features

The genomic features of *Lactiplantibacillus plantarum* CRS33 are summarized in Table 2. *Lactiplantibacillus plantarum* CRS33 possesses a single circular chromosome measuring 2,976,555 bp in length. The genomic DNA of *Lactiplantibacillus plantarum* CRS33 exhibits a GC (Guanine-Cytosine) content of 44.88% and encodes 2,806 protein-coding genes, in addition to 65S rRNA genes, 5 copies each of 16S and 23S rRNA genes, and 65 tRNA genes.

**Table 2:** Genome assembly and annotation of *Lactiplantibacillus plantarum* CRS33.

Features	Results
Genome size (bp)	2,976,555
GC content (%)	44.88
Coding genes	2,806
Gene total length (bp)	2,492,826
Gene/genome (%)	83.75
5S rRNA	6
16S rRNA	5
23S rRNA	5
tRNA	65
NR annotation	2,803
Swiss-Prot annotation	1,615
Pfam annotation	2,359
eggNOG annotation	2,355
GO annotation	2,265
KEGG annotation	1,405

### 3.2 Genomic functional annotation

**Figure 2:**
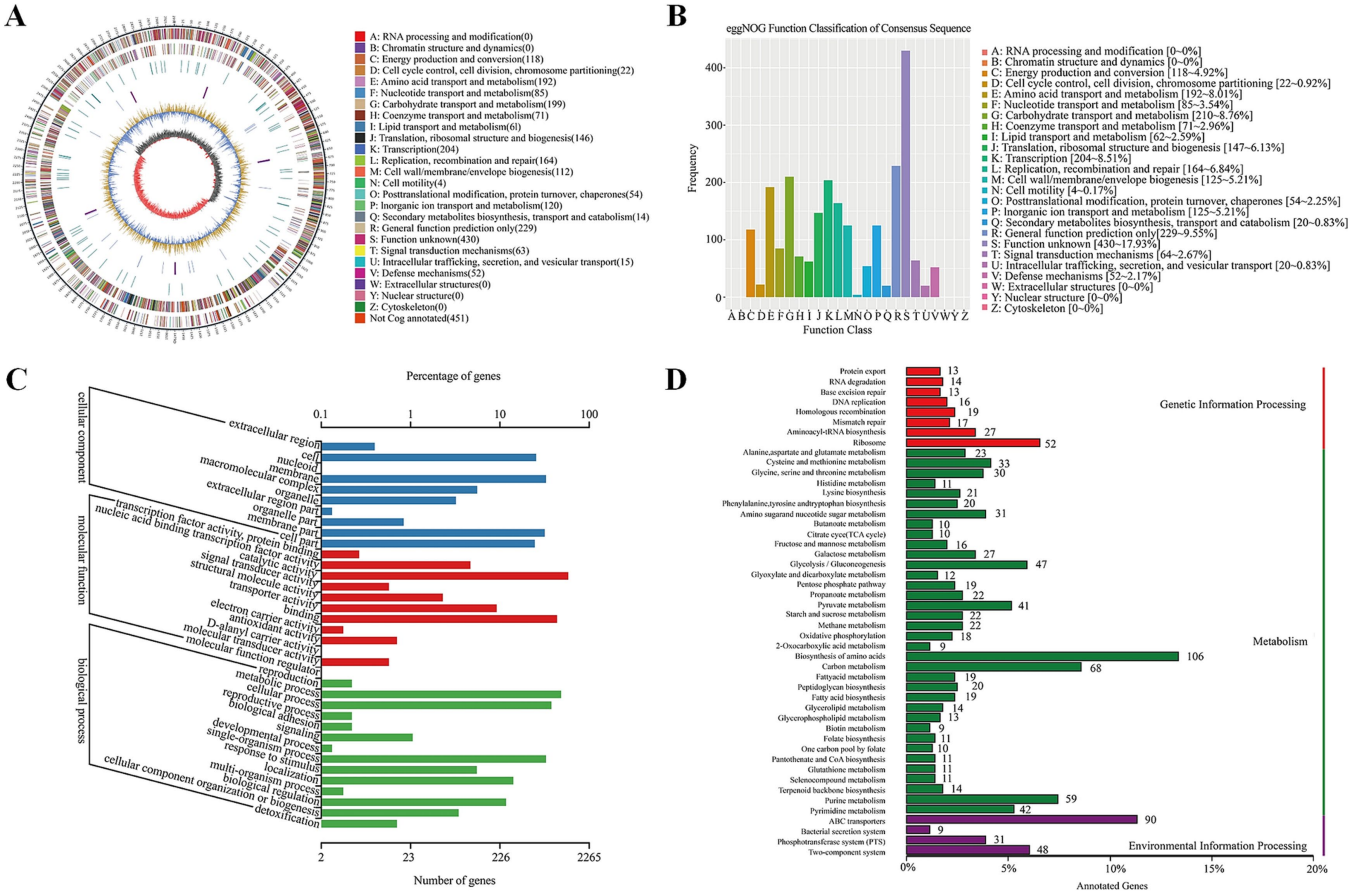
Whole-genome analysis of *Lactiplantibacillus plantarum* CRS33. **(A)** The circular genome representation of *Lactiplantibacillus plantarum* CRS33 is displayed. The outermost layer illustrates genome length, marked in 5-kb increments. The second and third layers depict genes located on the positive and negative strands, with color coding to show COG functional groups. The fourth layer represents repetitive regions, while the fifth layer identifies tRNA (blue) and rRNA (purple). The sixth layer illustrates GC composition, where light yellow indicates areas with GC composition above the genome-wide mean (higher peaks denote greater variation), and blue indicates regions below the average. The innermost layer represents GC-skew, where dark gray highlights G-enriched areas, and red highlights C-enriched areas. **(B)** EggNOG functional classification of proteins in the *Lactiplantibacillus plantarum* CRS33 genome; **(C)** GO classification of genes in the *Lactiplantibacillus plantarum* CRS33 genome; **(D)** KEGG pathway enrichment analysis for the *Lactiplantibacillus plantarum* CRS33 genome.

The genome of *Lactiplantibacillus plantarum* CRS33 was functionally annotated using multiple databases, such as NR, Swiss-Prot, Pfam, eggNOG, GO, and KEGG (Table 2). In total, 2,806 genes in the *Lactiplantibacillus plantarum* CRS33 genome underwent annotation. Of these, the majority were annotated in the NR (2,803 genes), Pfam (2,359 genes), eggNOG (2,355 genes), and GO (2,265 genes) databases. These annotations correspond to 99.87, 84.07, 83.93, and 80.72% of the total gene count, respectively. The Swiss-Prot and KEGG databases annotated fewer genes, identifying 1,615 and 1,405 genes, respectively. The circular genomic map of *Lactiplantibacillus plantarum* CRS33 is shown in Figure 2A.

Figure 2B illustrates the functional annotation results derived from the eggNOG database, categorized into 20 distinct functional groups. The most abundant category is carbohydrate transport and metabolism, followed by translation, amino acid transport and metabolism, and ribosomal structure and biogenesis. Furthermore, a Gene Ontology (GO) library analysis of *Lactiplantibacillus plantarum* CRS33 genes revealed 569 genes linked to cellular components (Figure 2C), 1,398 to molecular functions, and 298 to biological processes. The most prominent biological process-related genes were classified into metabolic activities, cellular activities, and single-organism activities. Moreover, *Lactiplantibacillus plantarum* CRS33 harbors genes associated with immune system functions and biological adhesion. Based on the KEGG database, the functional genes were categorized into 48 distinct classifications (Figure 2D), further grouped into three overarching categories: environmental information processing, genetic information handling, and metabolism. Among these, metabolism emerges as the most predominant category, with genes involved in amino acid biosynthesis being the most abundant. Moreover, genome annotation analysis identified numerous probiotic marker genes in *Lactiplantibacillus plantarum* CRS33, which are associated with antibacterial, anti-inflammatory, immunomodulatory, and antioxidant activities (Table 3). These genes play a crucial role in the probiotic effects of *Lactiplantibacillus plantarum* CRS33. A comparison with the CARD database confirmed that no resistance genes were present in the genome of *Lactiplantibacillus plantarum* CRS33.

**Table 3:** *Lactiplantibacillus plantarum* CRS33 genome antibacterial, anti-inflammatory pathway and related genes.

No.	Pathway ID	Description	Gene number
1	ko00072	Synthesis and degradation of ketone bodies	2
2	ko00121	Secondary bile acid biosynthesis	4
3	ko00130	Ubiquinone and other terpenoid-quinone biosynthesis	6
4	ko00362	Benzoate degradation	3
5	ko00401	Novobiocin biosynthesis	2
6	ko00430	Taurine and hypotaurine metabolism	5
7	ko00521	Streptomycin biosynthesis	3
8	ko00590	Arachidonic acid metabolism	1
9	ko00760	Nicotinate and nicotinamide metabolism	9
10	ko00900	Terpenoid backbone biosynthesis	14
11	ko01130	Biosynthesis of antibiotics	154

### 3.3 Effect of *Lactiplantibacillus plantarum* CRS33 on endometritis induced by *Escherichia coli*

To assess the impact of *Lactiplantibacillus plantarum* CRS33 in alleviating symptoms of endometritis, the mouse model of *E. coli*-induced endometritis was established. Compared with the CONT group, the uterine appearance of *E. coli* group exhibited obvious congestion and edema (Figure 1B). The uterine index in the *E. coli* group was markedly elevated compared to that of the CONT group (*p* < 0.01). In contrast, the LAB+*E. coli* and DEX + *E. coli* groups exhibited a considerable reduction in uterine index relative to the *E. coli* group (*p* < 0.01) (Figure 1D). Histological examination further revealed that the uterine tissue in the *E. coli* group exhibited hyperemia, edema, cellular degeneration, and extensive infiltration of inflammatory cells. In contrast, the LAB+*E. coli* and DEX + *E. coli* groups showed a uniform distribution of endometrial glands and significantly reduced inflammatory cell infiltration (Figure 1C).

### 3.4 Effect of *Lactiplantibacillus plantarum* CRS33 on inflammatory cell infiltration by *Escherichia coli* in the uterus

**Figure 3:**
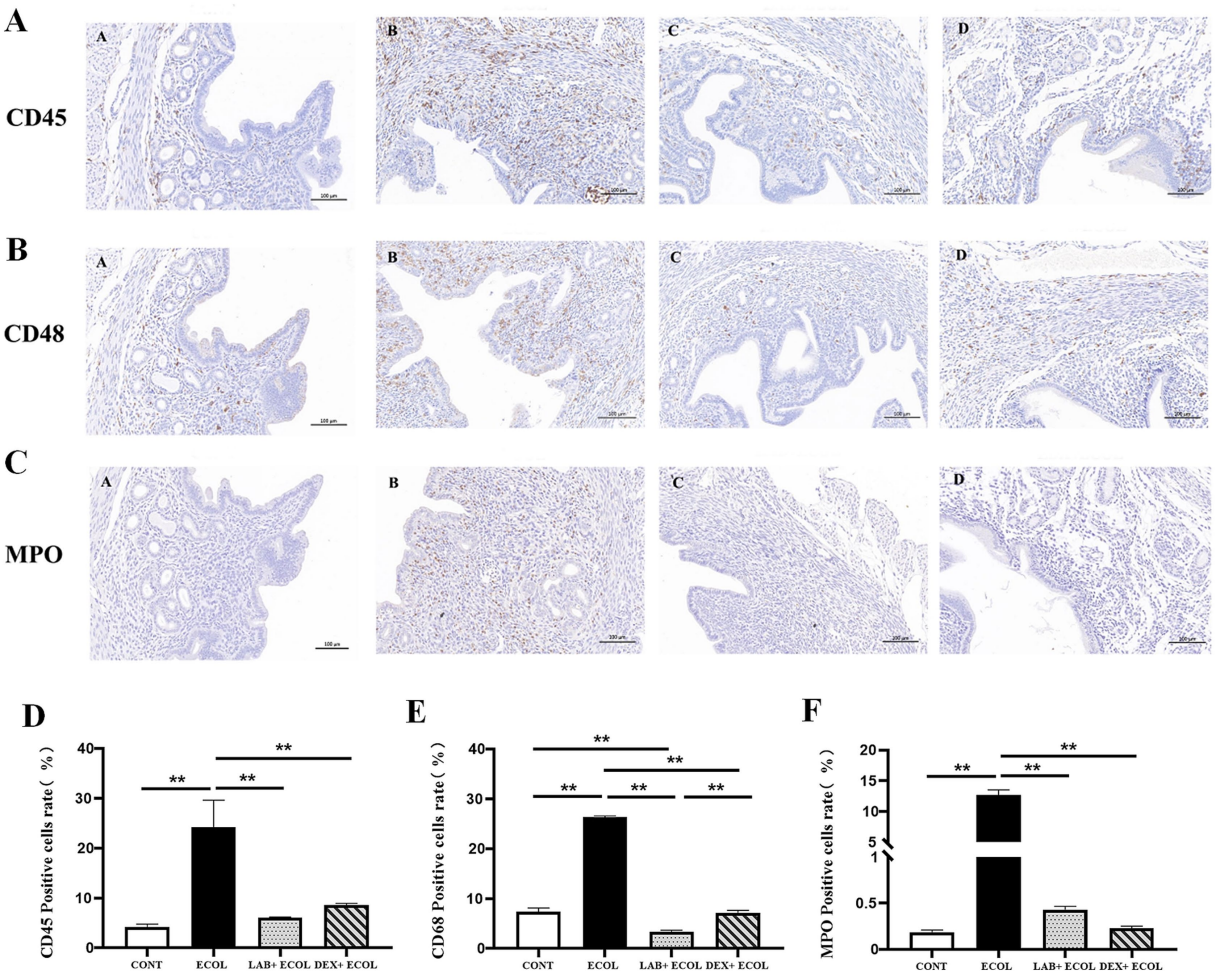
**(A–C)** Immunohistochemistry of mice uterine tissue (200×); **(D–F)** Expression of CD45, CD68, and MPO positive rates in mice uterus. *p < 0.05, **p < 0.01.

The activation and release of neutrophils, leukocytes, and macrophages serve as key drivers of tissue inflammation. The total number of leukocytes, macrophages and neutrophils were investigated using pan-leukocytes marker CD45, CD68 and MPO, respectively (Figures 3A–C). Uterine tissues in the *E. coli* group showed a significantly increased proportion of CD45, CD68, and MPO positive cells relative to the CONT group (*p* < 0.01). In contrast, the LAB+*E. coli* and DEX + *E. coli* groups showed a significantly reduced proportion of CD45, CD68, and MPO positive cells relative to the *E. coli* group (*p* < 0.01) (Figures 3D–F).

### 3.5 Effect of *Lactiplantibacillus plantarum* CRS33 on *Escherichia coli*-induced secretion of inflammatory factors in mouse uterine tissues

**Figure 4:**
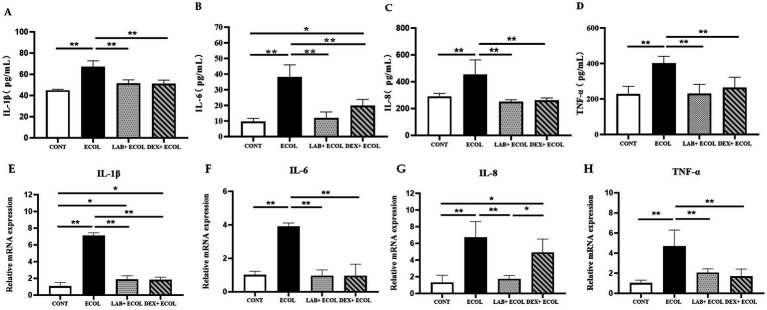
Effect of *Lactiplantibacillus plantarum* CRS33 on the secretion of inflammatory factors in *E. coli*-induced mice uterine tissues. **(A)** IL-1β concentrations; **(B)** IL-6 concentrations; **(C)** IL-8 concentrations; **(D)** TNF-*α* concentrations; **(E)** Relative IL-1β mRNA expression; **(F)** Relative IL-6 mRNA expression; **(G)** Relative IL-8 mRNA expression; **(H)** Relative TNF-α mRNA expression. The results are expressed as the mean ± SEM from three independent experiments. *p < 0.05, **p < 0.01.

Using ELISA, the secretion quantities of inflammatory cytokines were measured to determine the impact of *Lactiplantibacillus plantarum* CRS33 combined with dexamethasone on *E. coli*-induced inflammation in mouse uterine tissues (Figures 4A–D). The *E. coli* group showed notably increased levels of inflammatory markers relative to the CONT group (*p* < 0.01). However, inflammatory cytokines levels were markedly reduced in the LAB+*E. coli* and DEX + *E. coli* groups compared with the *E. coli* group (*p* < 0.01). Specifically, IL-6 levels were significantly lower in the LAB+*E. coli* group than in the DEX + *E. coli* group (*p* < 0.05). Meanwhile, no statistical differences were observed for IL-1β, IL-8, and TNF-*α* between these two groups (*p* > 0.05).

Compared to the CONT group, the *E. coli* group displayed a obvious rise in the mRNA expression of four key pro-inflammatory cytokines (IL-6, IL-8, IL-1β, and TNF-α) (*p* < 0.01). On the other hand, the LAB+*E. coli* and DEX + *E. coli* groups showed a significant decrease in the mRNA levels of these cytokines relative to the *E. coli* group (*p* < 0.01). Furthermore, no statistically significant differences were observed in the mRNA expression of IL-6 and TNF-α among LAB+*E. coli* and DEX + *E. coli* groups (*p* > 0.05) (Figures 4E–H).

### 3.6 Modulation of *Lactiplantibacillus plantarum* CRS33 in the endometrial microbiota composition in the *Escherichia coli*-induced endometritis mice

**Figure 5:**
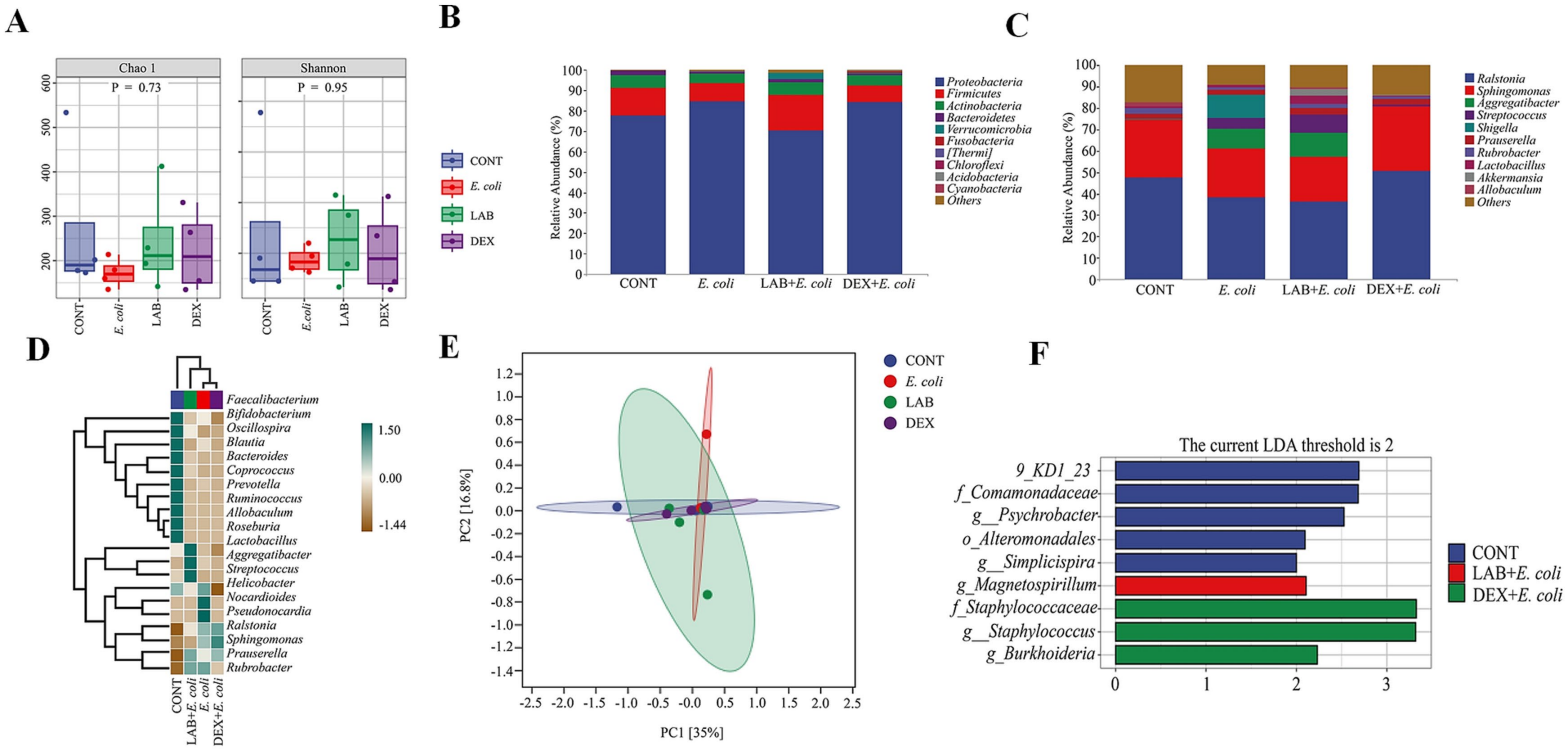
Changes in taxonomic composition of microorganisms in the uterus. **(A)** Alpha diversity index; **(B)** Taxonomic composition at the phylum level; **(C)** Taxonomic composition at the genus level; **(D)** Heat map of uterine microbial communities at the genus level; **(E)** OPLS-DA plot of uterine microorganisms; **(F)** LDA analysis.

To assess the impact of *Lactiplantibacillus plantarum* CRS33 on the composition of the endometrial microbiota in mice with endometritis, the Chao1 and Shannon indices were applied to measure the alpha diversity of the microbial community. Compared to the CONT group, the *E. coli* group displayed a considerable decrease in richness and alpha diversity. In contrast, the LAB+*E. coli* and DEX + *E. coli* groups significantly enhanced the microbial alpha diversity and richness in the uterine tissues of mice (Figure 5A).

In order to further analyze the influence of *Lactiplantibacillus plantarum* CRS33 on the profiles of the endometrial microbiota in mice with endometritis, the phylum abundance was examined. The results showed that the endometrial microbiota of each group of mice consisted mainly of *Proteobacteria*, *Firmicutes*, *Actinobacteria*, and *Bacteroidetes*, of these, *Proteobacteria* were the most dominant groups. Compared to the CONT group, the *E. coli* group displayed a simultaneous decline in the proportional abundance of *Firmicutes*, *Actinobacteria*, and *Bacteroidetes*, accompanied by a significant increase in *Proteobacteria*. The LAB+*E. coli* group exhibited a remarkable rise in the proportional abundance of *Firmicutes*, *Actinobacteria*, and *Bacteroidetes*, alongside a concurrent decrease in *Proteobacteria* within the LAB+*E. coli* group (Figure 5B). At the genus level, in comparison with the CONT group, the *E. coli* group showed obvious growth in the prevalence of *Shigella* and *Flexispira*, whereas the LAB+*E. coli* group demonstrated a concurrent reduction in their prevalence (Figure 5C).

Linear discriminant analysis (LDA) was deployed to determine the impact of individual species abundance on differential effects to discern significant groups or species responsible for sample delineation (Figure 5F). Significant differences were noted regarding the endometrial microbiota among the four groups. It showed that *Comamonadaceae*, *Psychrobacter* and *Alteromonadales* were identified as the dominant genera in CONT group. *Staphylococcaceae*, *Staphylococcus* and *Burkholderia* were as the dominant genera in the DEX + *E. coli* group, whereas *Magnetospirillum* was identified as the dominant genera in the LAB+*E. coli* group (Figures 5D,E).

## 4 Discussion

Genomic analysis can pinpoint genes linked to probiotic characteristics, such as those involved in cellular adhesion and the synthesis of antibacterial substances. In this research, we demonstrated that *Lactiplantibacillus plantarum* CRS33 has the ability to synthesize antimicrobial agents with broad-spectrum activity against pathogens. GC skew analysis provides insights into transcription direction and helps identify the coding strand in prokaryotic genomes, whereas GC content is commonly linked to genome stability (39, 40). Microbiome analysis can be affected by contamination risks and sequencing bias, which may impact the accuracy of the results. Contamination can occur at various stages of the process, from sample collection to DNA extraction and sequencing, potentially introducing external DNA and skewing the microbial profile, especially in low-biomass samples like the uterine microbiome (41). Additionally, sequencing bias can arise during PCR amplification, where certain microbial DNA is over-amplified, leading to a distorted community composition (42). To minimize these technical biases, it is recommended to use mock communities as controls, optimize DNA extraction and amplification procedures, and validate findings using multiple sequencing platforms (43–46). The GC skew and GC content of *Lactiplantibacillus plantarum* CRS33 in this research were found to be within the same range as those observed in other *Lactiplantibacillus plantarum* (47). Comprehensive comparative genomic analyses are imperative for further evaluating the safety and distinct probiotic characteristics of *Lactiplantibacillus plantarum* CRS33. GO, KEGG, and COG annotations revealed genes linked to carbohydrate metabolism, membrane transport, translation, and nucleotide metabolism, as illustrated in global and overview pathway maps. Gene analysis identified numerous common carbohydrate and amino acid metabolism-related genes in *Lactiplantibacillus plantarum* CRS33, suggesting its potential to modulate the host intestinal microbiota and promote health by influencing carbohydrate metabolism. Amino acids, within metabolic pathways, serve as fundamental building blocks of proteins and are essential for energy conversion and protein synthesis (48). Previous studies have demonstrated that glutamine is metabolized by enzymes like glutamine synthetase and glutamate dehydrogenase to produce energy. Furthermore, tryptophan catabolism plays a pivotal role in regulating kynurenine-mediated immune processes during infections, inflammation, and pregnancy (49, 50). This study revealed that the genome of *Lactiplantibacillus plantarum* CRS33 harbors lipid metabolism-related genes, suggesting its potential involvement in modulating host lipid metabolism. Probiotics support the host by synthesizing or releasing specific bioactive compounds through diverse metabolic pathways.

Previous studies suggest that probiotics confer benefits to the host by synthesizing or releasing specific bioactive compounds through metabolic activities (51). The genome of *Lactiplantibacillus plantarum* CRS33 encodes numerous genes linked to antibacterial, anti-inflammatory, and immune-regulatory pathways. For instance, *Lactiplantibacillus plantarum* CRS33 harbors genes involved in antibiotic biosynthesis pathways, targeting the synthesis of neomycin and streptomycin, thereby inhibiting or eliminating pathogenic microorganisms. Additionally, *Lactiplantibacillus plantarum* CRS33 encodes genes responsible for the biosynthesis of ubiquinone and related terpenoid-quinone compounds. Extensive research highlights that terpenoids regulate the NF-κB pathway and exhibit substantial therapeutic potential against inflammation and cancers (52). The genome of *Lactiplantibacillus plantarum* CRS33 also encodes pathways for niacin and nicotinamide metabolism. Niacin, also known as vitamin B3, functions as a lipid-modulating agent widely used in treating severe chronic inflammatory conditions. Upon conversion to nicotinamide (NAM), niacin serves as a precursor for NAD + synthesis, thereby stimulating SIRT1 activation. This mechanism suppresses the NF-κB signaling pathway, reduces the production of pro-inflammatory mediators, and exerts anti-inflammatory effects (53, 54). Secondary bile acids, derive d from primary bile acids via bacterial metabolism in the intestine, inhibit harmful bacterial growth, maintain intestinal flora balance, and modulate immune responses by activating nuclear receptors like FXR and TGR5 (55, 56). Similarly, taurine and hypotaurine, non-essential amino acids found in humans and animals, participate in diverse metabolic processes, including antioxidation, anti-inflammation, and cellular protection (57). Through the synthesis of various metabolic products mentioned above, *Lactiplantibacillus plantarum* CRS33 exhibits effective antibacterial, antioxidant, anti-inflammatory, and immune-modulating properties. Resistance genes acquired by probiotic strains are recognized as a major concern for the potential spread of antimicrobial resistance among different bacterial species (58). Notably, no resistance genes were detected in the genome of *Lactiplantibacillus plantarum* CRS33, suggesting a minimal risk of antimicrobial resistance dissemination. Nevertheless, safety aspects relevant to application in cattle—such as uterine colonization dynamics, persistence/clearance, and interactions with the native reproductive microbiome—have not yet been evaluated and warrant dedicated assessment prior to clinical translation.

*E. coli*, a prevalent Gram-negative bacterium, is regarded as the primary pathogen associated with bovine endometritis (59). It activates the uterine immune defense system, triggering inflammation, immune cell infiltration, and pathological changes in uterine tissue (60, 61). The vaginal microbiota predominantly composed of *Lactobacillus* species is essential for managing and preventing bovine uterine inflammation (62). In this study, *Lactiplantibacillus plantarum* CRS33 present an excellent therapeutic effect on mice endometritis through reducing endometrial inflammation, attenuating the inflammatory response, regulating diversifies the microbial community and increased the proportion of the dominant flora in the uterus.

However, it is important to note that while these results are promising, the mouse model used in this study has inherent limitations. Physiological differences between mice and dairy cows—including uterine anatomy, estrous cycling, and postpartum immune dynamics—mean that outcomes observed in mice may not fully translate to cattle. In addition, the absence of bovine clinical validation restricts the direct applicability of these findings to real-world dairy production. Despite these limitations, the mouse model offers clear advantages that make it a reasonable preclinical proxy to a limited extent: it provides highly controlled and reproducible experimental conditions, faster study timelines, and lower costs; it enables rigorous randomization/blinding and standardized dosing/routes; and it leverages a rich toolkit of immunological reagents to probe mechanisms relevant to inflammation across mammals. Thus, our murine data should be viewed as hypothesis-generating evidence that prioritizes *Lactiplantibacillus plantarum* CRS33 for targeted evaluation in cattle, rather than as definitive proof of efficacy in dairy cows.

The release of neutrophils, leukocytes, and macrophages is the primary catalyst for tissue inflammation (63). The total number of leukocytes, macrophages and neutrophils were investigated using pan-leukocytes marker CD45, CD68 and MPO, respectively (64). Our research demonstrated that the activities of CD45, CD68, and MPO were markedly elevated in the *E. coli* group relative to the CONT group (*p* < 0.01). Nevertheless, treatment with *Lactiplantibacillus plantarum* CRS33 and dexamethasone led to a significant reduction in CD45, CD68, and MPO levels. In agreement with earlier findings, *Lactobacillus* has been shown to lower CD45 (65), CD68 (66), and MPO (67) levels in damage caused by inflammation. This treatment restored the immune balance of uterine inflammatory cells (CD45, CD68, MPO) in mice. Additionally, *Lactiplantibacillus plantarum* CRS33 reduced uterine tissue swelling and inflammatory infiltration, consistent with the observed reduction in leukocytes, macrophages, and neutrophils. The pronounced infiltration of neutrophils into and around the endometrial glands might be associated with increased levels of inflammatory cytokines, which are key mediators in initiating phagocytic activity at sites of inflammation (68). In our study, the levels of these inflammatory cytokines were significantly elevated in the uterine tissue of mice infected with *E. coli*. However, the administration of *Lactiplantibacillus plantarum* CRS33 led to a marked reduction in these cytokines. Earlier research has demonstrated that *Lactobacilli* derived from the vaginal microbiota of dairy cows can effectively suppress the secretion of inflammatory cytokines (69), which is consistent with our findings. Thus, *Lactiplantibacillus plantarum* CRS33 shows potential as an anti-inflammatory agent in treating *E. coli*-induced endometritis.

The endometrial microbiome is essential to regulate the reproductive process. The dysbiosis of endometrial microbiome is associated with uterine diseases (70). The structure and activity of the microbial community are key factors in the progression of endometritis (71). In this research, treatment with *Lactiplantibacillus plantarum* CRS33 enhanced the proportions of *Firmicutes*, *Actinobacteria*, and *Bacteroidetes*, while reducing the prevalence of *Proteobacteria*. Additionally, *Proteobacteria* have been identified as primary bacteria implicated in *E. coli*-induced endometritis. Consistent with previous studies, increased levels of *Proteobacteria* were observed in sows with endometritis, compared to their healthy counterparts (72). At the genus level, our findings revealed that *E. coli* infection notably elevated the presence of *Shigella* and *Flexispira*. In the LAB+*E. coli* group, *Akkermansia* and *Lactobacillus* were more abundant. Moreover, administration of *Lactiplantibacillus plantarum* CRS33 resulted in a reduction of *Bifidobacterium* and *Allobaculum*. These bacteria, such as *Akkermansia*, *Lactobacillus*, *Bifidobacterium*, and *Allobaculum*, are beneficial microorganisms known to exert direct or indirect anti-inflammatory effects (73–75). The findings suggest that *Lactiplantibacillus plantarum* CRS33 mitigates endometritis in mice by promoting the proliferation of anti-inflammatory bacteria. However, studies have shown that probiotics can improve health outcomes not only by directly exerting antimicrobial effects but also by modulating the host microbiome, which in turn affects immune responses and inflammation (76). Thus, while the direct antimicrobial and anti-inflammatory properties of *Lactiplantibacillus plantarum* CRS33 are evident, the ecological changes in the uterine microbiota could also play a role in mitigating *E. coli*-induced endometritis. Conversely, the levels of these beneficial bacteria in the uterine microbiota were notably reduced in the DEX + *E. coli* group compared to the LAB+*E. coli* group. This suggests that *Lactiplantibacillus plantarum* CRS33 exhibits stronger anti-inflammatory effects than dexamethasone in addressing *E. coli*-induced endometritis in mice. Notably, the predominant bacterial family in the dexamethasone-treated group was *Staphylococcaceae*. Members of *Staphylococcus*, especially coagulase-negative strains, are well known for producing bacteriocins that exhibit antimicrobial properties against *E. coli* (77). Therefore, it is hypothesized that dexamethasone treatment contributed to the increased abundance of *Staphylococcaceae* in the uterine microbiota of mice with endometritis. Furthermore, *Lactobacillus sali*var*ius* antagonizes *E. coli* by stabilizing the intestinal microbiome composition (78), aligning with our results. Therefore, our study concludes that *Lactiplantibacillus plantarum* CRS33 can mitigate *E. coli*-induced endometritis in mice by modulating the diversity and balance of the uterine microbiota.

## 5 Conclusion

In this study, *Lactiplantibacillus plantarum* CRS33 showed promising potential in mitigating the inflammatory response caused by *E. coli* infection in mice with endometritis. This was achieved through its synthesis of various antibacterial and anti-inflammatory compounds, as well as its ability to suppress inflammatory cell infiltration and reduce the secretion of inflammatory mediators. Additionally, *Lactiplantibacillus plantarum* CRS33 appeared to modulate the composition and structure of the uterine microbial community disrupted by *E. coli*, contributing to the restoration of microbial diversity and abundance. However, these findings are preliminary and should be interpreted as proof-of-concept. Further validation studies in large animal models, such as cattle, are needed before considering clinical applications.

## Data Availability

The original contributions presented in our study are publicly available. The data can be found at the following link: National Center for Biotechnology Information/CP125654.1.

## Footnotes

^1^ https://www.genescloud.cn
