# Supplementary material for: Evaluation of Lactiplantibacillus plantarum CRS 33 to therapeutic effects on a murine model of Escherichia coli-induced endometritis
Source: Front Vet Sci. 2025 Oct 31;12:1608791. doi: 10.3389/fvets.2025.1608791 (PMC12616865; doi:10.3389/fvets.2025.1608791)
Supplement: Supplementary file 1 [file Data_Sheet_1.zip › Data Sheet 1 (5)/Supplementary Data Sheet 1_captions.docx]

**Supplementary Data Sheet 1:**

**Supplementary Data Sheet 1 - Figure 1**: Immunohistochemical Staining and Positive Cell Rates for CD45, CD48, and MPO

**Captions**

a. Immunohistochemical staining for CD45 in various experimental groups (A: Control, B: ECOL, C: LAB+ECOL, D: DEX+ECOL). Representative images showing CD45-positive cells, with scale bars indicating 100 µm.

b. Immunohistochemical staining for CD48 in various experimental groups (A: Control, B: ECOL, C: LAB+ECOL, D: DEX+ECOL). Representative images showing CD48-positive cells, with scale bars indicating 100 µm.

c. Immunohistochemical staining for MPO in various experimental groups (A: Control, B: ECOL, C: LAB+ECOL, D: DEX+ECOL). Representative images showing MPO-positive cells, with scale bars indicating 100 µm.

Column Diagram: Bar graphs depicting the positive cell rates of CD45 (A), CD68 (B), and MPO (C) in different experimental groups. Data are presented as the percentage of positive cells, with statistical significance indicated by asterisks (p < 0.01).

Scale Immunohistochemistry: Original images of the immunohistochemical staining used to create the composite images shown in immunohistochemistry. These images provide the scale for each staining type, with original magnifications and scaling factors indicated in the images.

**Supplementary Data Sheet 1 - Figure 2**: Histological Sections of Mice Uterine Tissue (100× Magnification, H&E Staining)

**Captions**

Figure 1-4: Hematoxylin and eosin (H&E) staining of a section of mouse uterine tissue. The image shows the structural details of the uterine lining and underlying tissues at 100× magnification. A scale bar of 200 µm is provided for reference.

**Raw Data Files**

The remaining files—labeled "Elisa," "immunohistochemistry," "pcr," and "wb"—contain the original raw data for the bar charts presented in the manuscript. These data include results from the respective assays used to generate the figures and graphs in the text.
