# Supplementary figures and images for: Evaluation of Lactiplantibacillus plantarum CRS 33 to therapeutic effects on a murine model of Escherichia coli-induced endometritis
Source: Front Vet Sci. 2025 Oct 31;12:1608791. doi: 10.3389/fvets.2025.1608791 (PMC12616865; doi:10.3389/fvets.2025.1608791)

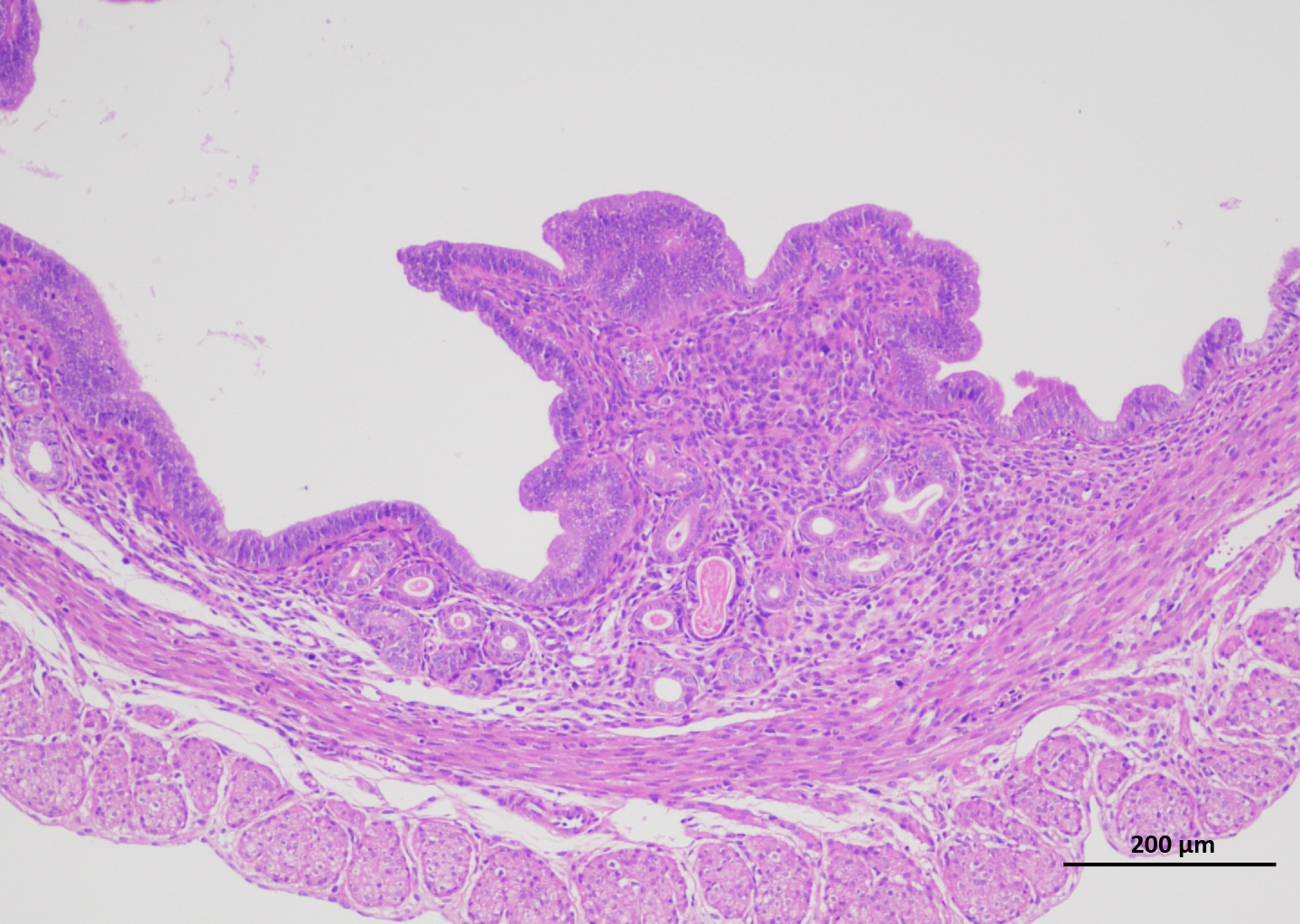

Supplement: Supplementary file 1 [file Data_Sheet_1.zip › Data Sheet 1 (5)/Figure 1/A.jpg]

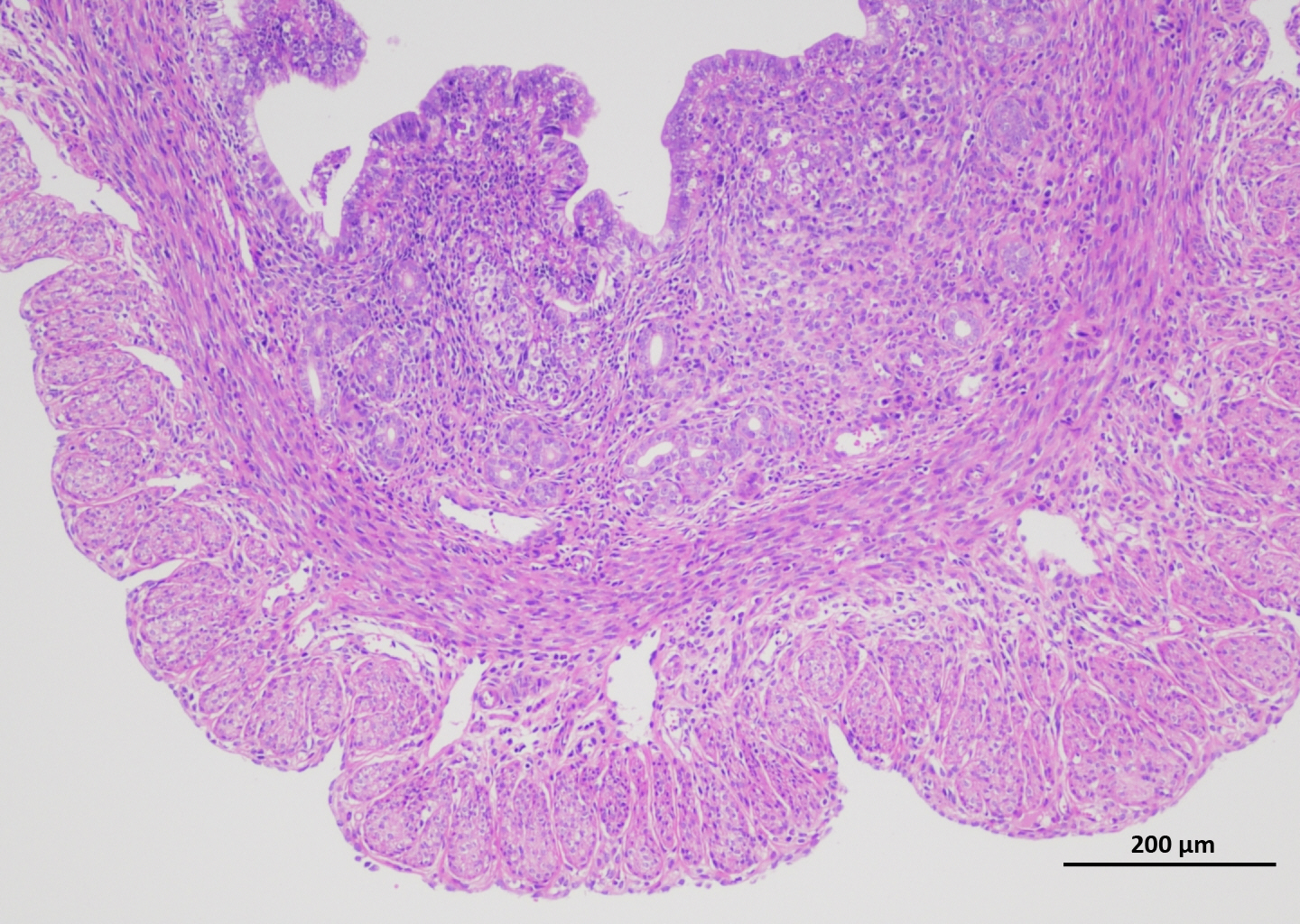

Supplement: Supplementary file 1 [file Data_Sheet_1.zip › Data Sheet 1 (5)/Figure 1/B.jpg]

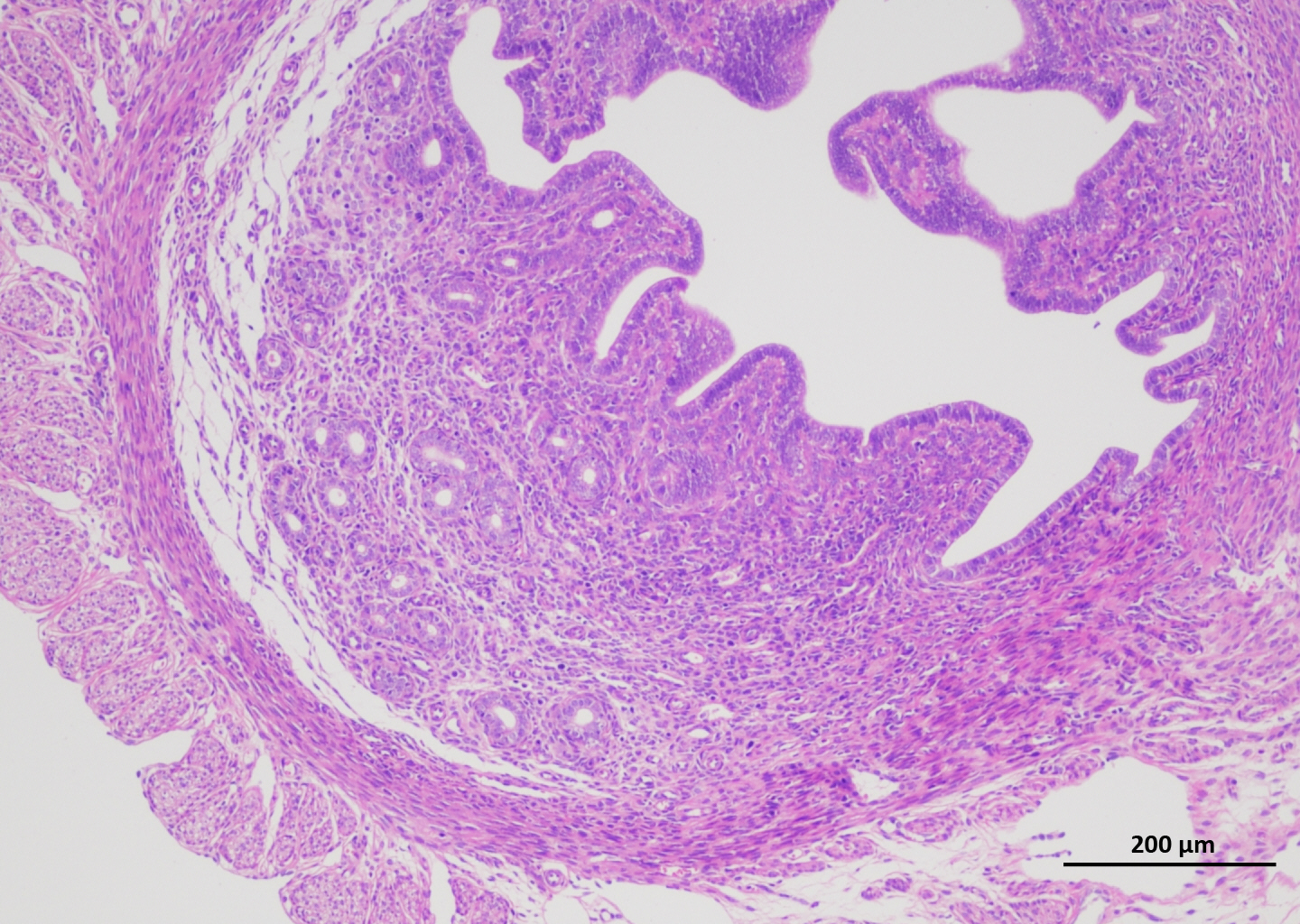

Supplement: Supplementary file 1 [file Data_Sheet_1.zip › Data Sheet 1 (5)/Figure 1/C.jpg]

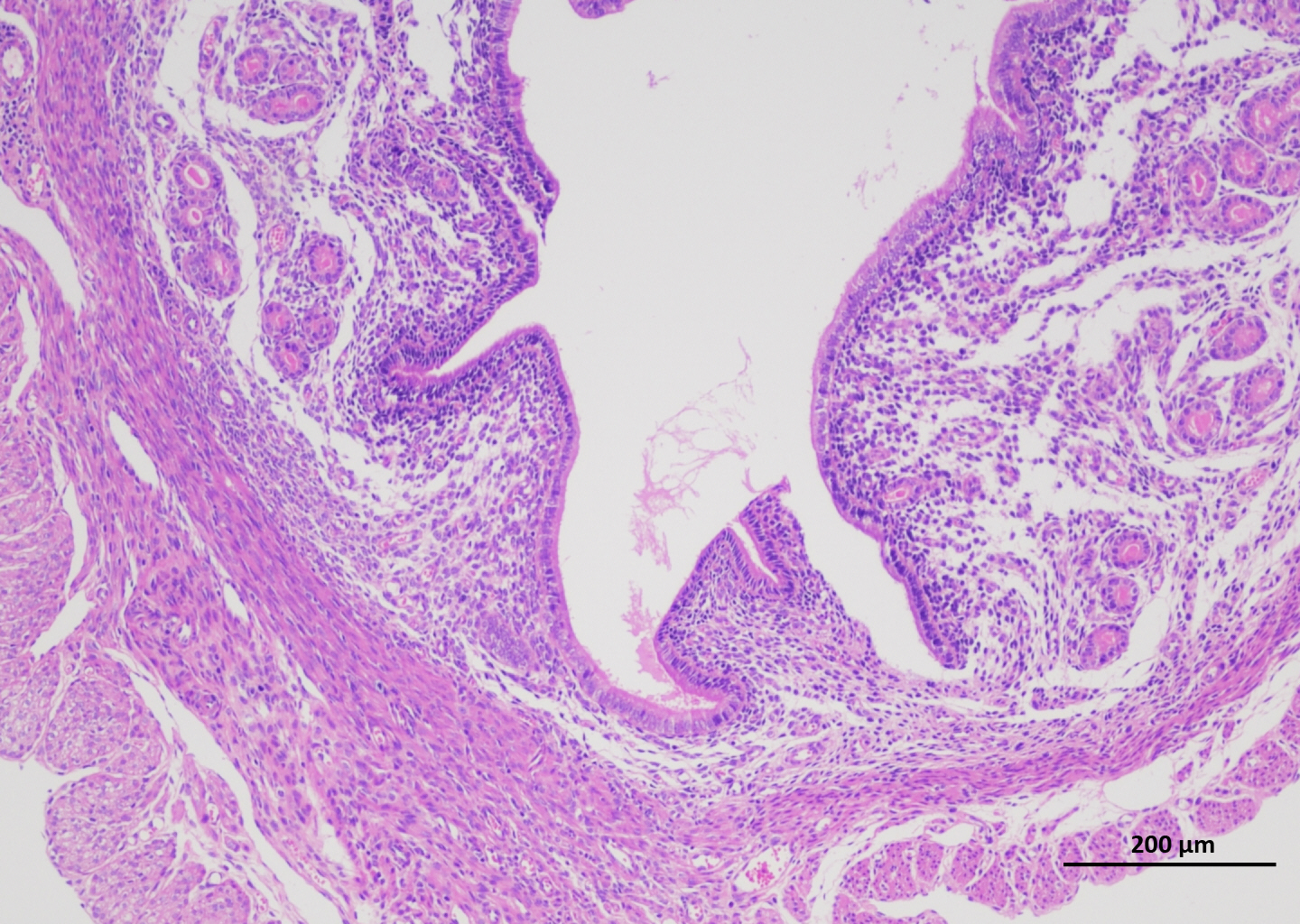

Supplement: Supplementary file 1 [file Data_Sheet_1.zip › Data Sheet 1 (5)/Figure 1/D.jpg]

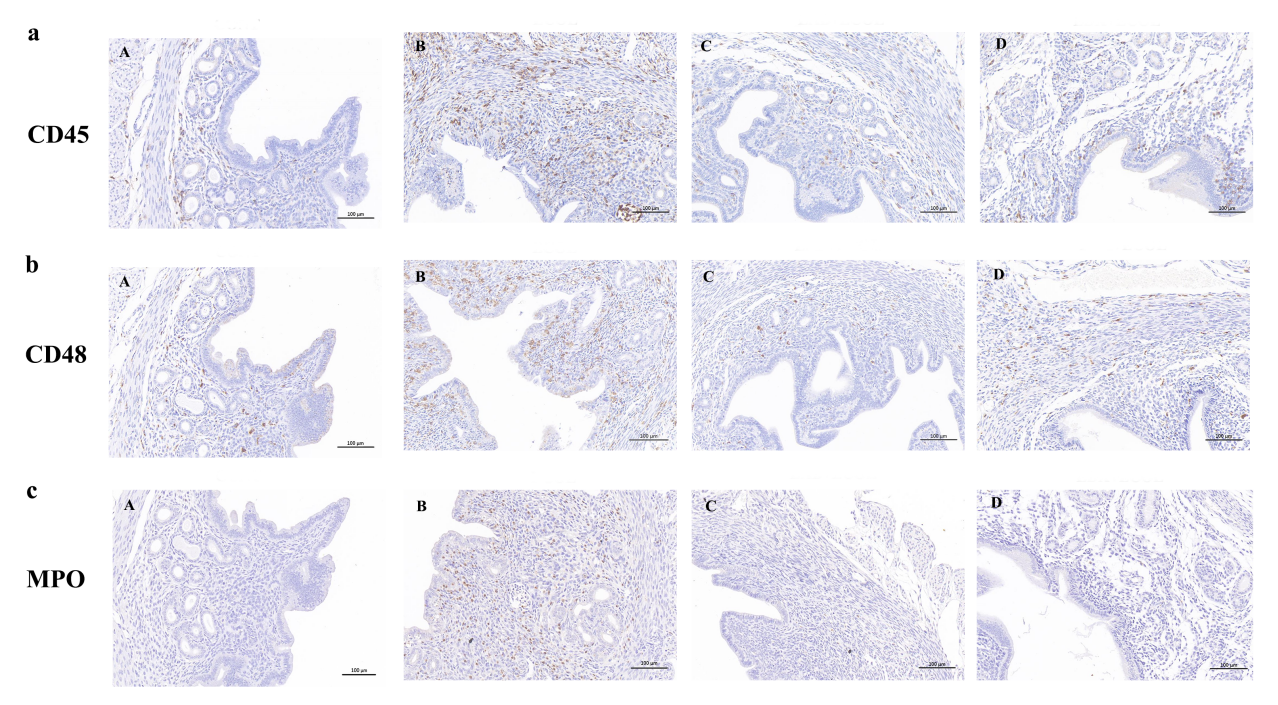

Supplement: Supplementary file 1 [file Data_Sheet_1.zip › Data Sheet 1 (5)/Figure 2/immunohistochemistry/免疫组化.png]

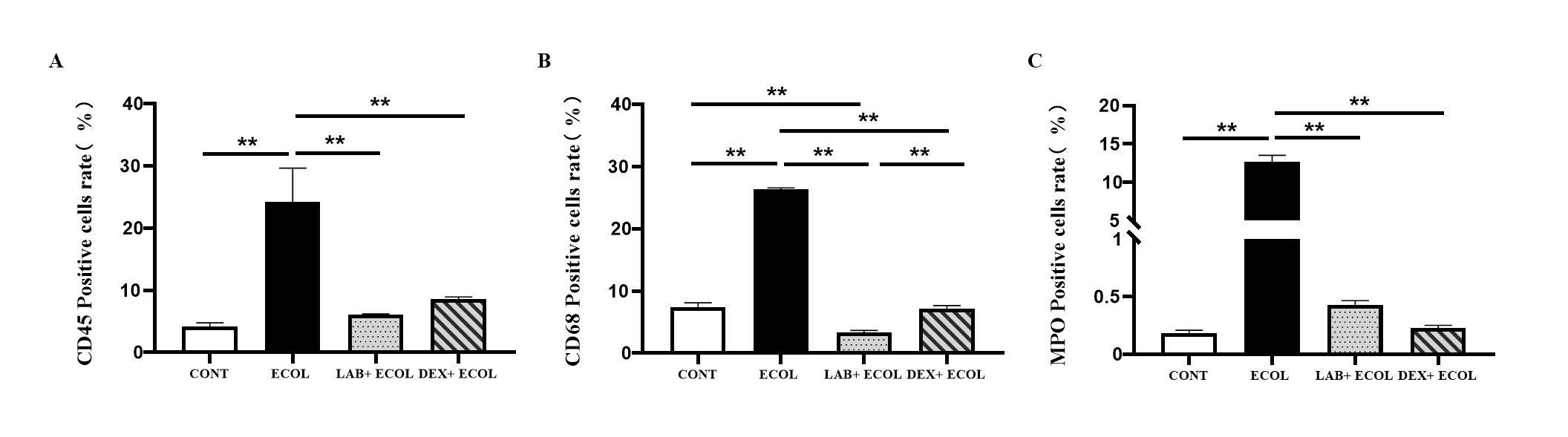

Supplement: Supplementary file 1 [file Data_Sheet_1.zip › Data Sheet 1 (5)/Figure 2/immunohistochemistry/免疫组化柱状图.png]

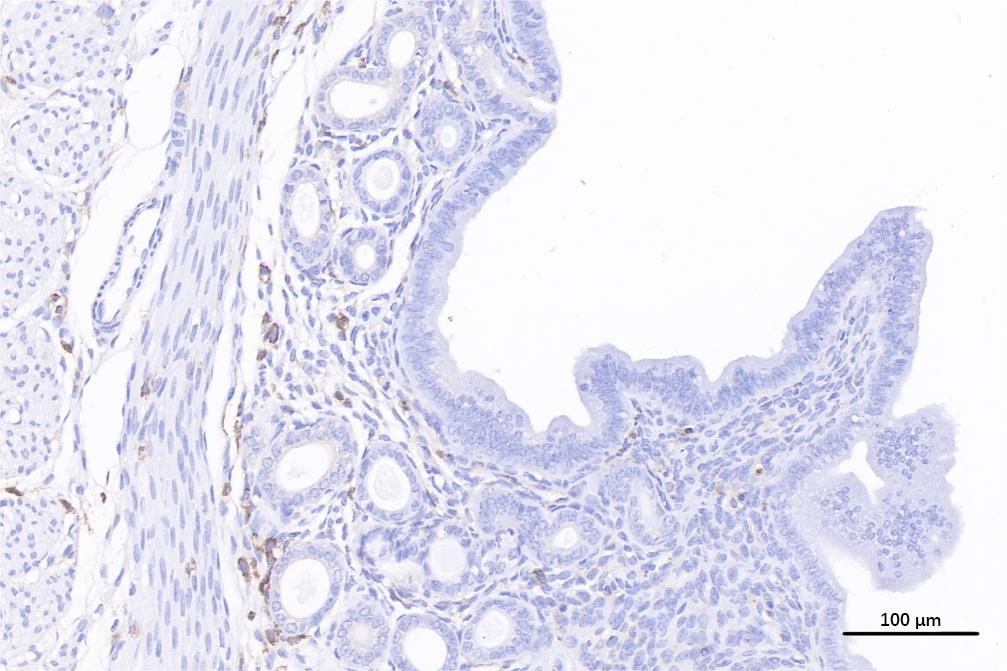

Supplement: Supplementary file 1 [file Data_Sheet_1.zip › Data Sheet 1 (5)/Figure 2/Scale immunohistochemistry/标尺 CD45/A CONT(1).png]

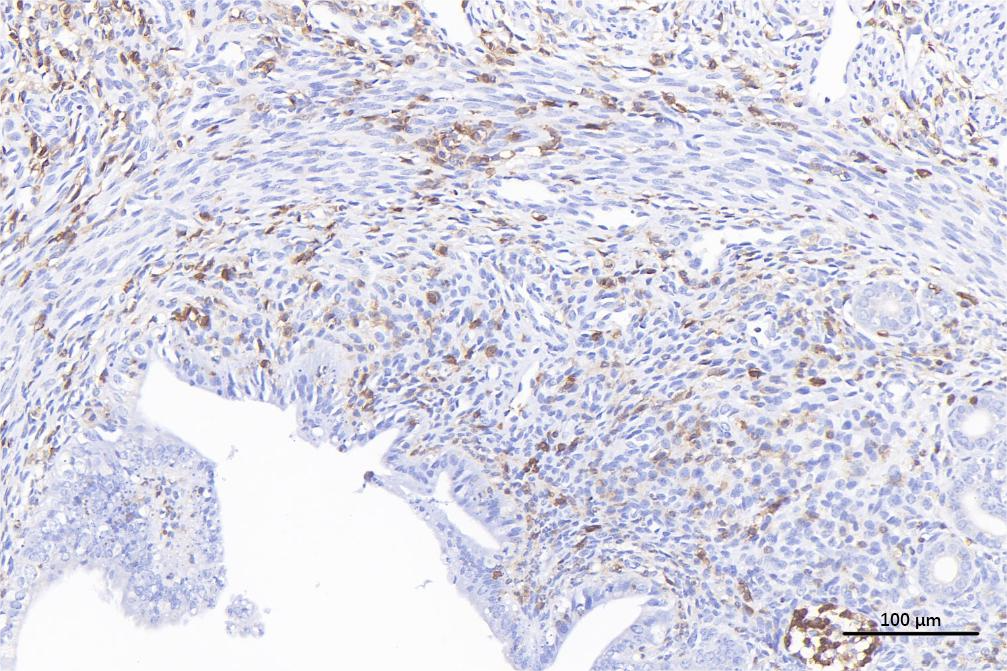

Supplement: Supplementary file 1 [file Data_Sheet_1.zip › Data Sheet 1 (5)/Figure 2/Scale immunohistochemistry/标尺 CD45/B ECOL(1).png]

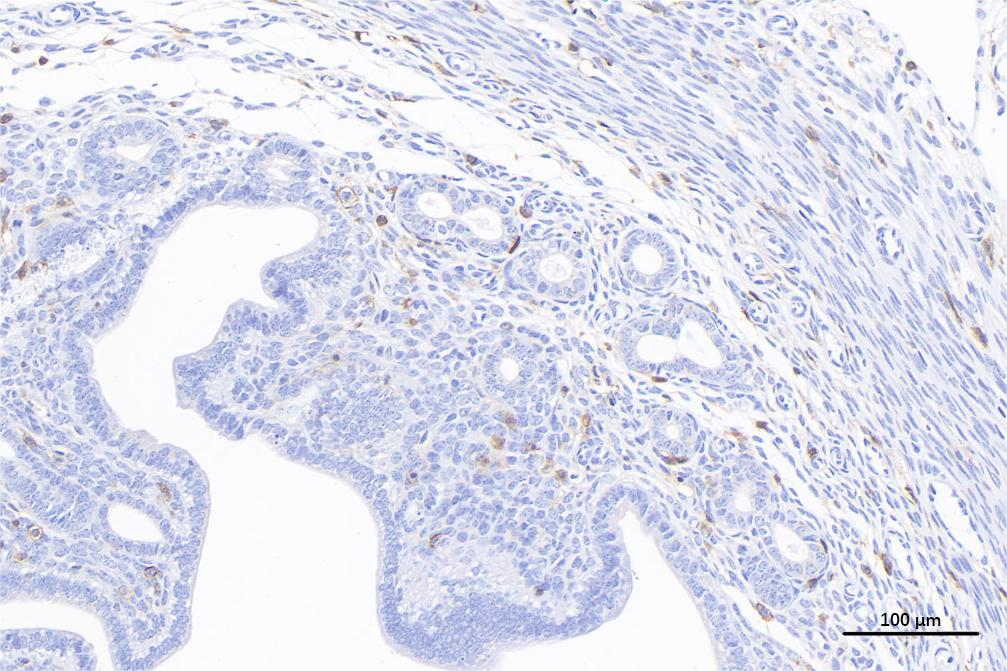

Supplement: Supplementary file 1 [file Data_Sheet_1.zip › Data Sheet 1 (5)/Figure 2/Scale immunohistochemistry/标尺 CD45/C LAB+ECOL(1).png]

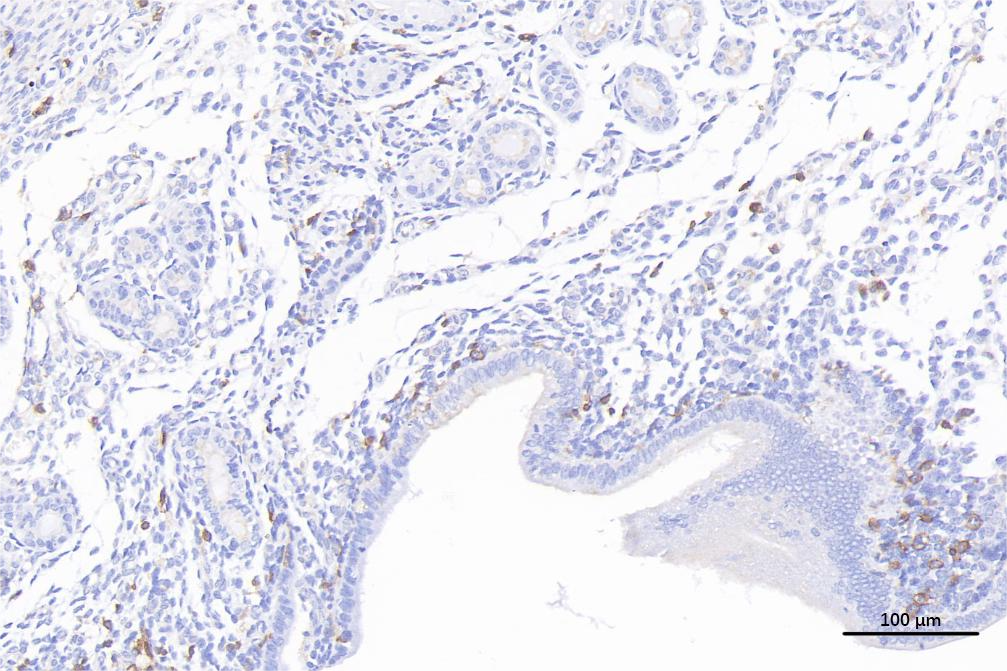

Supplement: Supplementary file 1 [file Data_Sheet_1.zip › Data Sheet 1 (5)/Figure 2/Scale immunohistochemistry/标尺 CD45/D DEX+ECOL(1).png]

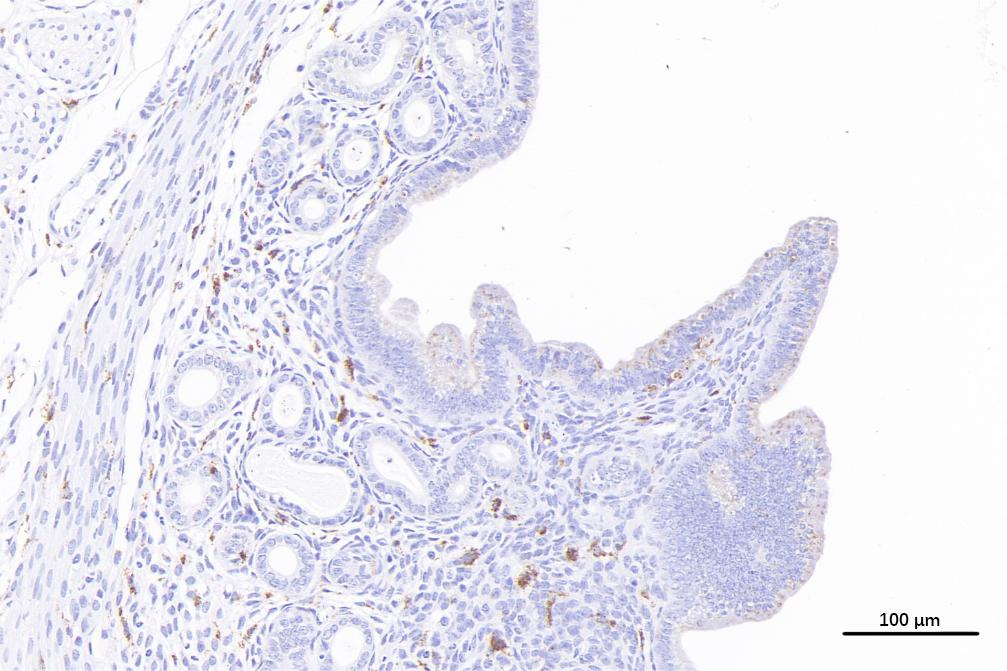

Supplement: Supplementary file 1 [file Data_Sheet_1.zip › Data Sheet 1 (5)/Figure 2/Scale immunohistochemistry/标尺 CD86/A CONT(1).png]

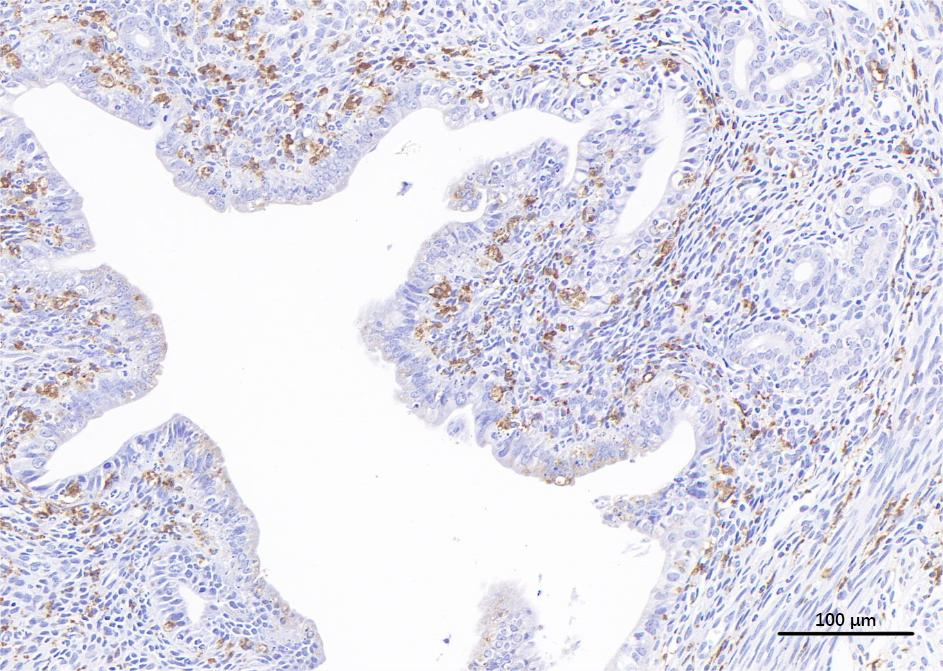

Supplement: Supplementary file 1 [file Data_Sheet_1.zip › Data Sheet 1 (5)/Figure 2/Scale immunohistochemistry/标尺 CD86/B ECOL(1).png]

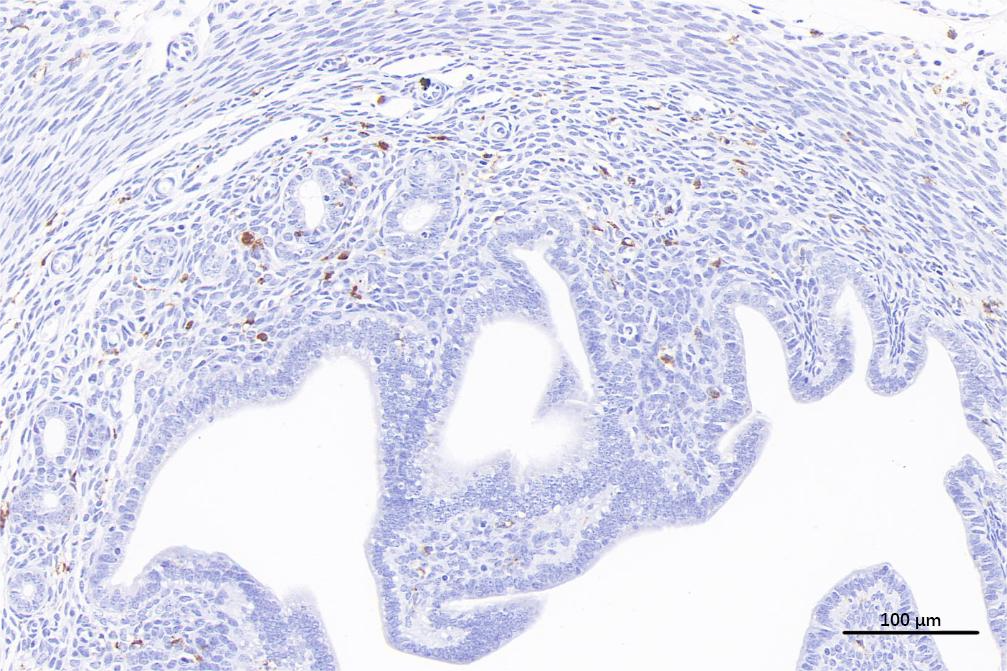

Supplement: Supplementary file 1 [file Data_Sheet_1.zip › Data Sheet 1 (5)/Figure 2/Scale immunohistochemistry/标尺 CD86/C LAB+ECOL(1).png]

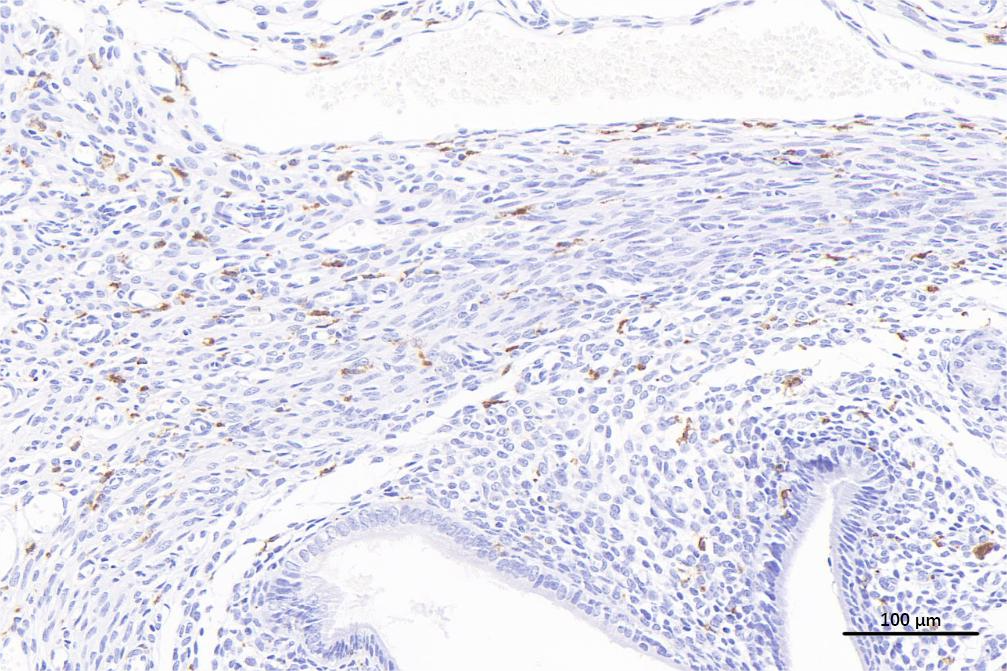

Supplement: Supplementary file 1 [file Data_Sheet_1.zip › Data Sheet 1 (5)/Figure 2/Scale immunohistochemistry/标尺 CD86/D DEX+ECOL(1).png]

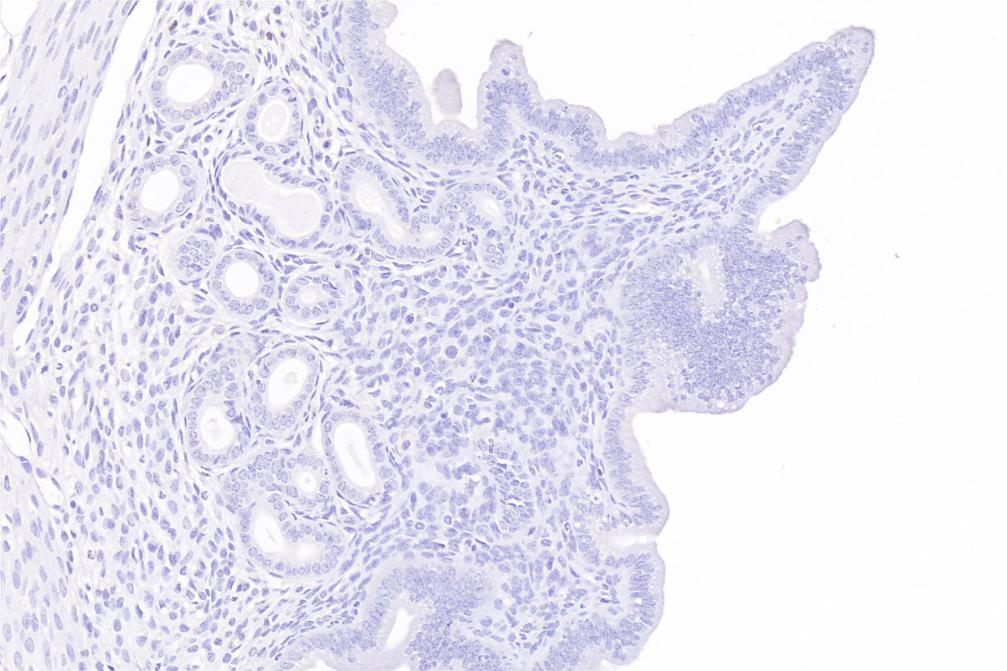

Supplement: Supplementary file 1 [file Data_Sheet_1.zip › Data Sheet 1 (5)/Figure 2/Scale immunohistochemistry/标尺 MPO/A CONT(1).png]

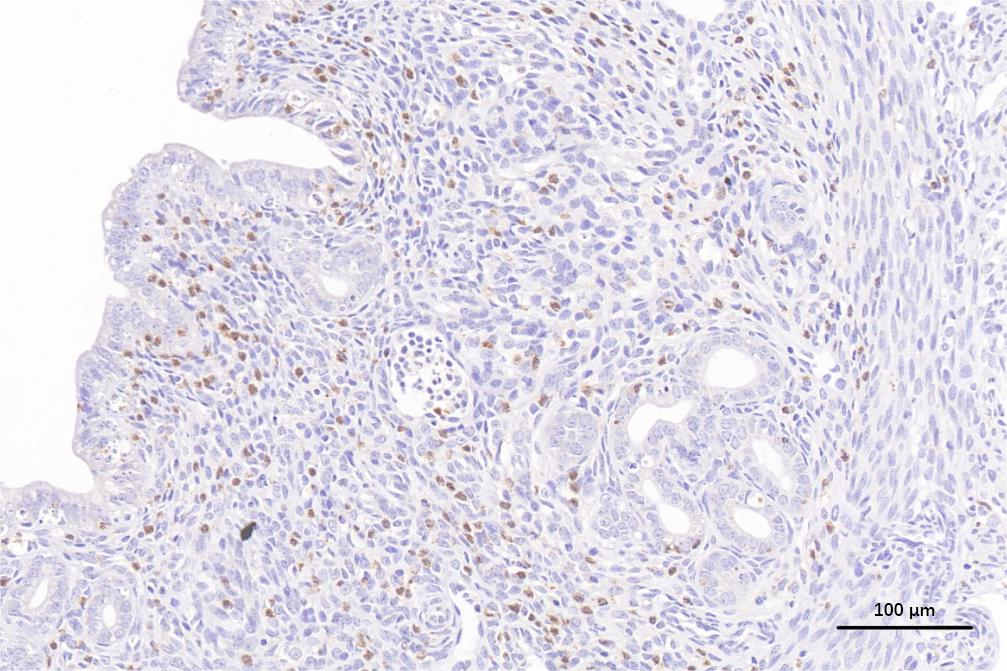

Supplement: Supplementary file 1 [file Data_Sheet_1.zip › Data Sheet 1 (5)/Figure 2/Scale immunohistochemistry/标尺 MPO/B ECOL(1).png]

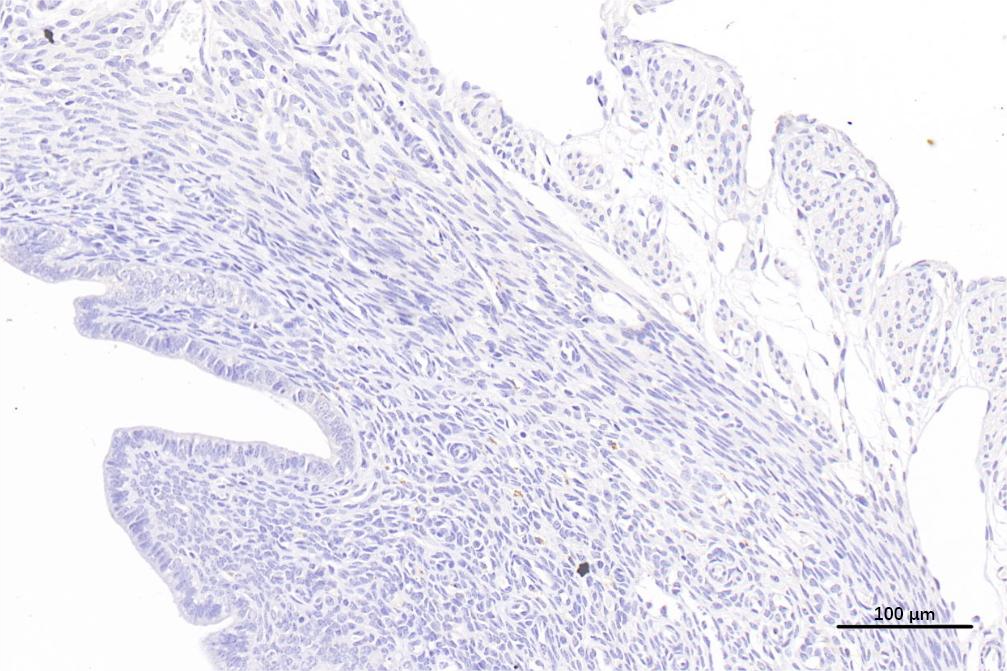

Supplement: Supplementary file 1 [file Data_Sheet_1.zip › Data Sheet 1 (5)/Figure 2/Scale immunohistochemistry/标尺 MPO/C LAB+ECOL(1).png]

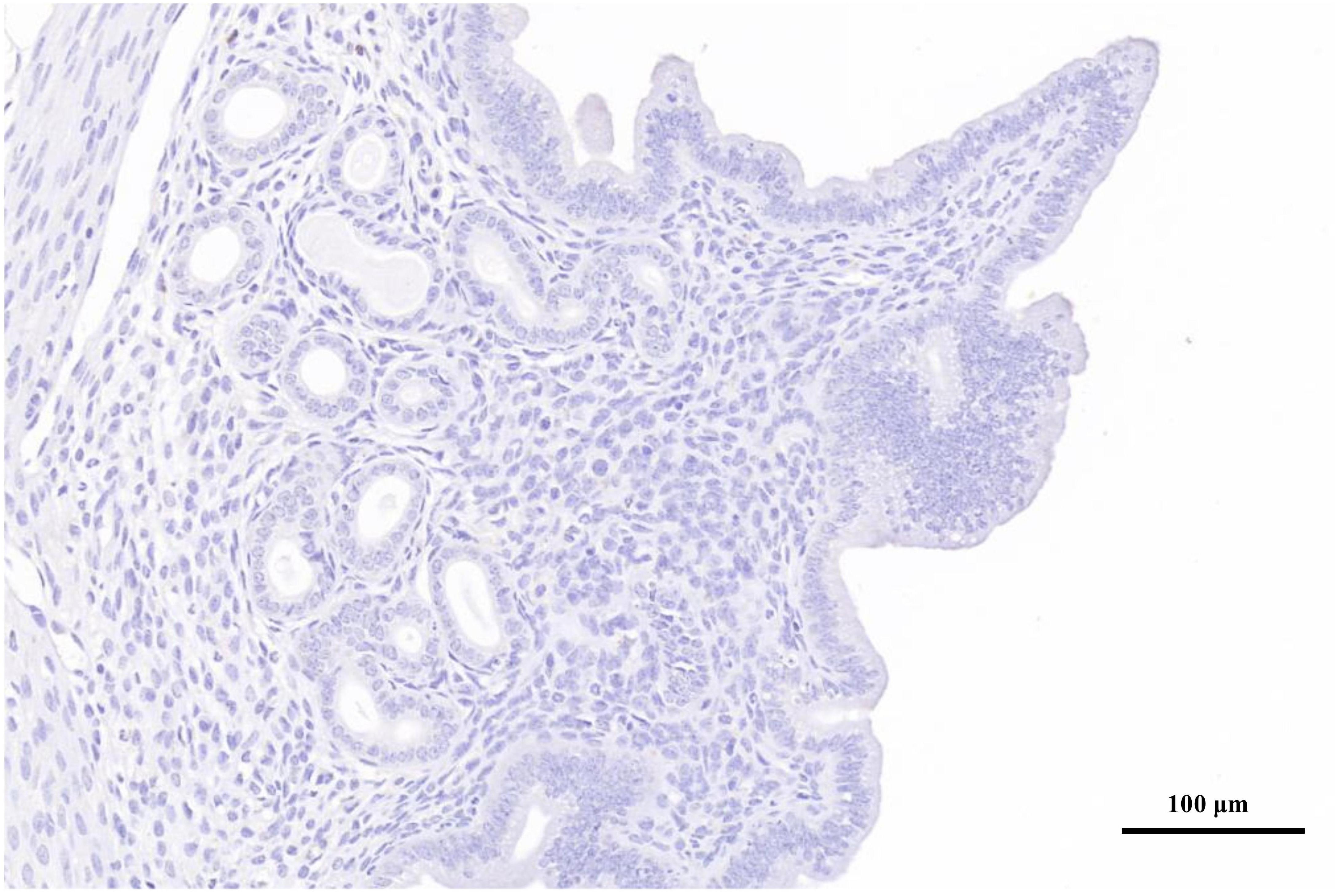

Supplement: Supplementary file 1 [file Data_Sheet_1.zip › Data Sheet 1 (5)/Figure 2/Scale immunohistochemistry/标尺 MPO/CONT.jpg]

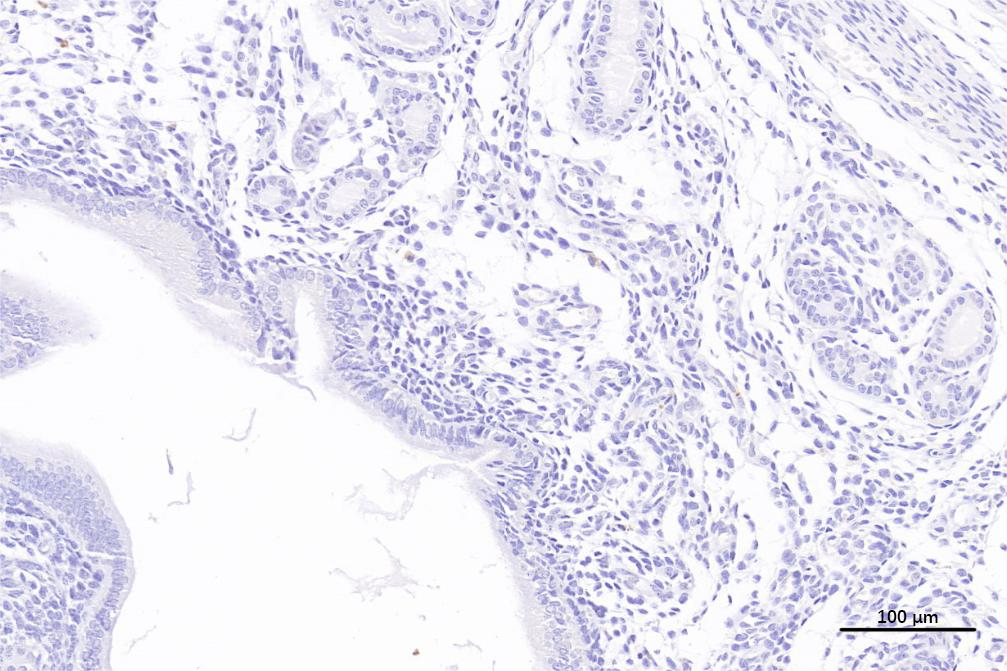

Supplement: Supplementary file 1 [file Data_Sheet_1.zip › Data Sheet 1 (5)/Figure 2/Scale immunohistochemistry/标尺 MPO/D DEX+ECOL(1).png]
